# Supplementary material for: Confidence Intervals for Adaptive Trial Designs I: A Methodological Review
Source: Stat Med. 2025 Aug 8;44(18-19):e70174. doi: 10.1002/sim.70174 (PMC12333484; doi:10.1002/sim.70174)
Supplement: Supplementary file 1 — Data S1. Supporting Information. [file SIM-44-0-s001.pdf]

Systematic review of CIs for ADs

Search term used in Scopus:
TITLE-ABS-KEY (("confidence interval" OR "confidence region" OR "confidence limit" OR "confidence band") AND ("adaptive design" OR "adaptive trial" OR "adaptive clinical trial" OR "group sequential" OR "sequential trial" OR "sequential clinical trial" OR " drop the loser" OR "response adaptive" OR "sample size re-estimation" OR "seamless phase" OR "multi arm multi stage" OR "adaptive enrichment" OR “master protocol” OR “platform trial”))

Link to reproduce the search:
https://www.scopus.com/results/results.uri?sort=plf-t&src=s&sid=bc60f028a238a35c1a95a06273f1ce96&sot=a&sdt=a&sl=440&s=TITLE-ABS-KEY%28%28%22confidence+interval%22+OR+%22confidence+region%22+OR+%22confidence+limit%22+OR+%22confidence+band%22%29+AND+%28%22adaptive+design%22+OR+%22adaptive+trial%22+OR+%22adaptive+clinical+trial%22+OR+%22group+sequential%22+or+%22sequential+trial%22+OR+%22sequential+clinical+trial%22+OR+%22+drop+the+loser%22+OR+%22response+adaptive%22+OR+%22sample+size+re-estimation%22+OR+%22seamless+phase%22+OR+%22multi+arm+multi+stage%22+OR+%22adaptive+enrichment%22+OR+%22master+protocol%22+OR+%22platform+trial%22%29%29

A “title, abstract, keywords” search on 7 April 2025 yielded 366 results, 219 of which can be excluded. The remaining 147 results are summarised in the table below.

#	Paper title & authors	Brief description of the methodology	Trial context and advantages / limitations	Code/software available?	Case studies?
**1**	The analysis of sequential clinical trials<br><br>Whitehead and Jones (1979)<br>https://doi.org/10.1093/biomet/66.3.443	Uses approach of Siegmund (1978), i.e. inverting hypothesis tests	Sequential tests with normally distributed observations, allowing for overshoot and underrunning<br><br>Only asymptotically valid (for large N and small value of treatment effect)	No	No

			Simulations show that the proposed test procedure is conservative		
**2**	Exact confidence intervals following a group sequential test<br><br>Tsiatis et al. (1984)<br>https://doi.org/10.2307/2530924	Final/exact CIs derived by inverting a family of two-sided hypothesis tests, using stage-wise ordering of the sample space	Group sequential designs with normally distributed responses<br><br>CIs based on stage-wise ordering can be used without knowledge of information levels beyond the observed stopping stage. However, does not always give a confidence interval containing the MLE.<br><br>CIs can only be calculated at the stage the trial stops, according to pre-specified stopping rules	No	No
**3**	Repeated confidence intervals for group sequential clinical trials<br><br>Jennison and Turnbull (1984)<br>https://doi.org/10.1016/0197-2456(84)90148-X	Widths of repeated intervals based on Pocock and O'Brien and Fleming boundaries.	Group Sequential designs, repeated confidence intervals for normal and survival data (intervals on hazard rate).<br><br>Computation is straightforward but these CIs are not tied to stopping rule (specifically stated there is no implication that they are). However, it is possible to construct corresponding tests.	No<br>(Possibly now available elsewhere)	No
**4**	Repeated confidence intervals for the median survival time<br><br>Jennison and Turnbull (1985)<br>https://doi.org/10.1093/biomet/72.3.619	Use asymptotic results shown first in the single stage. This is then extended to the multi-stage problem by treating as a series of experiments of increasing size.	Group sequential, survival data. Can be used in the setting of right censored observations assuming independent competing risks.<br><br>Results are given for fixed analysis times and constant increase in accrual. Like	No<br>(Possibly now available elsewhere)	No

			above error rates for associated tests can be computed. This requires increments to be equal but suggestion that it may be robust to variations in group size. (other work suggests otherwise)		
**5**	Interim analyses in randomized clinical trials: ramifications and guidelines for practitioners<br><br>Geller and Pocock (1987)<br>https://www.jstor.org/stable/2531962	Review of final/exact CIs (Tsiatis et al. 1984) and repeated CIs (Jennison and Turnbull, 1984)	Final/exact CIs " make no correction when a trial is stopped after the first analysis, which is problematic"<br><br>RCIs maintain type I error probability for any interval but "if one's primary interest is in<br>a final confidence interval after stopping, this approach is unduly conservative"	No	No
**6**	Confidence intervals following group sequential tests<br><br>Chang and O'Brien (1986)<br>https://doi.org/10.1016/0197-2456(86)90004-8	Sample space ordered based on likelihood principle. CIs based on likelihood ratio test.	Group sequential trials, binomial outcomes. They note that they found the confidence set was not always a continuous interval but state the effect of this was negligible.	Fortran available upon request. (So likely as the above)	No
**7**	Confidence intervals following group sequential tests in clinical trials<br><br>Kim and DeMets (1987)<br>https://doi.org/10.2307/2531539	Extension of (2) to allow for sample sizes to differ between stages (or analyses to be unequally timed).<br>Only after trial.	Group sequential, normally distributed. Results are not exact (not derived from asymptotics but simulation). Usable with error spending tests.<br>Extension to survival analysis possible (would be achieved as in (4)).	No	No

**8**	Exact confidence intervals following a group sequential trial: A comparison of methods<br><br>Rosner and Tsiatis (1988)<br>https://doi.org/10.1093/biomet/75.4.723	Final CIs using idea of ordering the sample space.	Group sequential. Intervals are dependent on the ordering chosen. Exact coverage following the termination of a group sequential test. The discussion is useful as to when they suggest each ordering.	No	No
**9**	Confidence intervals for a normal mean following a group sequential test<br><br>Chang (1989)<br>https://doi.org/10.2307/2532050	Based on likelihood ratio test, objective to produce smaller intervals than Tsiatis et al. (1984). This is the same idea as Chang and O'Brien (1986) but applied to normal data.<br><br>Single interval after test.	Group sequential, normally distributed data. No guarantee that the MLE falls in the confidence interval (stated as almost always according to experience).	No	No
**10**	Statistical approaches to interim monitoring of medical trials: A review and commentary<br><br>Jennison and Turnbull (1990)<br>https://doi.org/10.1214/ss/1177012099	A summary of repeated confidence intervals work and discussion around the context. Includes discussion of controversy around these points (specifically that they are wider than those computed only after a rejection).	NA	NA	NA
**11**	An improved approximation for calculation of confidence intervals after a sequential clinical trial	Removes continuous monitoring assumption. Focus is on the triangular test. Confidence intervals are	Group sequential, relying only on asymptotic normality.	Yes (PEST2.0 noted in paper)	No

	Facey and Whitehead (1990)<br>https://doi.org/10.1002/sim.4780091107	computed after the conclusion of the trial.	CI is tied to the crossing of the stopping boundary.<br><br>An important note in general from the paper. They state how other authors have considered the width of CIs in terms of optimality. However due to asymmetry this may be misleading.		
**12**	Statistical inference with data-dependent treatment allocation rules<br><br>Wei et al. (1990)<br>https://www.tandfonline.com/doi/abs/10.1080/01621459.1990.10475319	Derives exact conditional CIs for the odds ratio and difference in proportions, by inverting CDF of the number of successes. Conditional CI is conditional on the number of patients allocated to treatment and the total number of successes.<br>Unconditional CI based on methods of Santner and Snell (1980) and Suissa and Shuster (1985)<br><br>Also proposes approximate CIs based on 1) normal approximation of test statistics and 2) profile likelihood based chi-squared approximation	RPW designs<br><br>Unconditional intervals are much shorter than conditional intervals<br><br>Approximate CIs based on normal approximation perform poorly for small and moderate-sized samples (coverage far below nominal levels). Profile-likelihood based CIs perform quite well for N > 50	No	Re-analysis of RAR trial
**13**	Interim analyses with repeated measurements in a sequential clinical trial	Repeated measures with sequential stopping rule. Analyses are carried out at	In terms of confidence intervals this follows the repeated confidence interval approach.	No	No

	Wei et al. (1990)<br>https://doi.org/10.1093/biomet/77.2.359	calendar times with a regression model used at each analysis. From here the group sequential test is inverted and the repeated confidence interval approach may be used.			
**14**	Parameter estimation following group sequential hypothesis testing<br><br>Emerson and Fleming (1990)<br>https://doi.org/10.1093/biomet/77.4.875	Define confidence intervals by the "acceptance region" approach (seen elsewhere) for non-extreme results for the sufficient statistic (again relying on ordering)	Estimation following a group sequential hypothesis test for the mean of a normal distribution with known variance is investigated<br><br>A proposed ordering of the sample space based on the maximum likelihood estimate of the mean is found to result in estimates which compare favourably with estimates computed from orderings investigated by Tsiatis, Rosner & Mehta (1984) and Chang & O'Brien (1986) for a variety of group sequential designs	No	No
**15**	Exact statistical inference for group sequential trials<br><br>Lin et al. (1991)<br>https://doi.org/10.2307/2532394	Uses repeated confidence interval approach applied to group sequential trials with binomial outcomes	Group sequential, binomial. Repeated confidence intervals and following confidence intervals.<br><br>Approach recommended for small or moderate sized trials.	No	No
**16**	Exact calculations for sequential t, x and f tests	An extension of the repeated confidence intervals methods to the associated distributions.	Follows the repeated confidence interval approach.	No	No

	Jennison and Turnbull (1991)<br>https://doi.org/10.1093/biomet/78.1.133				
**17**	Repeated confidence intervals and prediction intervals using stochastic curtailment<br><br>Davis and Hardy (1992)<br>https://doi.org/10.1080/03610929208830783	An extension of the repeated confidence intervals methods to incorporate stochastic curtailment	For use with stochastic curtailment procedures for two samples in the estimation of differences of means, differences of proportions, odds ratios, and hazard ratios	No	Re-analyses data from three group sequential trials
**18**	Exact repeated confidence intervals for Bernoulli parameters in a group sequential clinical trial<br><br>Coe and Tamhane (1993)<br>https://doi.org/10.1016/0197-2456(93)90047-H	CIs found by grid search such that P(l<T<u) as nominal coverage	Single arm and two-arm sequential designs with binary outcomes<br><br>Exact coverage (up to grid search size)<br><br>Generalisation to RR and ORR indicated<br><br>Numeric search can be computationally intensive	BASIC and FORTRAN code form 1st author	Re-analyses data from a group sequential trial
**19**	Monitoring of clinical trials: Issues and recommendations	No specific methodology, but a review type article	Recommends that the "repeated confidence intervals" do not serve the same role as the fixed confidence intervals - the latter can be used for stopping rules but the magnitude	No	No

	Fleming and DeMets (1993)<br>https://doi.org/10.1016/0197-2456(93)90002-U		of the treatment effect should be reported using RCIs		
**20**	Sequential equivalence testing and repeated confidence intervals, with applications to normal and binary responses<br><br>Jennison and Turnbull (1993)<br>https://doi.org/10.2307/2532600	CIs found by inversion of sequential tests (2 different versions). This is the repeated CI approach	Equivalence testing problem with binary or normal data<br><br>Repeated confidence intervals, can cope with deviations from planned group sizes<br><br>RCIs sensitive to planning assumption of effect (like all equivalence tests)	No	No
**21**	Use of the randomization distribution in the analysis of sequential clinical trials<br><br>Snapinn (1994)<br>https://doi.org/10.1080/03610929408831268	Proposes the use of the randomisation distribution to calculate adjusted p-values by repeating the sequential analysis for each (or a random sample) of the possible random allocations of participants to arms and ordering the sample space based on stagewise ordering.<br><br>Obtains a CI by simply re-centering the naive CI at the adjusted point estimate.	Arbitrary sequential trials<br><br>CIs conceptually simple and applicable regardless of the type of boundary or distribution of the populations<br>CIs computationally intensive<br>CIs performs well (in terms of coverage) for small-to-moderate values of treatment effect but deteriorates with larger values	No	Re-analysis of data from CONSENSUS and CONSENSUS II trials

**22**	Interim monitoring of bivariate responses using repeated confidence intervals<br><br>Cook (1994)<br>https://doi.org/10.1016/0197-2456(94)90056-6	Joint sequential CIs found by inversion of sequential test. Builds on Jennison & Turnbull (1989) and (1993), i.e. Repeated CIs	Sequential superiority and equivalence tests with two parameters of interest. Derived for normal, but also illustrated for binary and TTE.<br><br>Potentially conservative as rectangular regions are found<br><br>Depends on good estimate of covariance matrix.	No	Hypothetical examples
**23**	A microcomputer program for the design and analysis of phase II cancer clinical trials with two group sequential methods, the sequential probability ratio test, and the triangular test<br><br>Bellissant et al. (1994)<br>https://doi.org/10.1006/cbmr.1994.1003	No new method but a computer implementation of:<br><br>Jones and Whitehead. Sequential forms of the logrank and modified Wilcoxon tests for censored data. Biometrika 66, 105 (1979).<br><br>and<br><br>Whitehead and Jones. The analysis of sequential clinical trials. Biometrika 66,<br>443 (1979).		Mentions code, but actual program does not seem available. From description probably defunct on most current systems anyway.	Illustrates computation on (what was then) an ongoing group sequential trial
**24**	A unified method for monitoring and analysing controlled trials	Construct a testing procedure based on usual z-test but penalize with additional	Normal data, 2 group comparison<br><br>- not yielding nominal coverage	No	Yes

	Grossman et al. (1994)<br>https://doi.org/10.1002/sim.4780131804	(hypothetical) data with mean difference of zero. Usual Wald intervals are constructed and called credibility intervals as no attempt is made for them to yield nominal coverage	- essentially a Bayesian approach but don't call it this.		
**25**	Monitoring of a pilot toxicity study with two adverse outcomes<br><br>Etzioni and Pepe (1994)<br>https://doi.org/10.1002/sim.4780132203	Constructs a confidence region for the frequencies of the adverse outcomes by simulating p-values and inverting the test procedure.<br><br>One of the few papers that propose a multivariate confidence region rather than just univariate confidence intervals	Preliminary single-arm toxicity study with bivariate binary adverse outcomes using a Bayesian posterior probability criterion for safety monitoring but frequentist inference at the end<br><br>- Computationally intensive	No	No
**26**	Group sequential monitoring of clinical trials with multivariate outcomes<br><br>Lee (1995)<br>https://doi.org/10.1177%2F00928615950290S106	Repeated CIs as in Jennison & Turnbull (1989)	Longitudinal and multivariate sequential superiority test	No	Re-analysis of group sequential trial data
**27**	Group sequential extensions of a standard bioequivalence testing	Usual Wald intervals are used and heuristically adapted to	Normal data in equivalence test setting	No	No

	procedure<br><br>Gould (1995)<br>https://link.springer.com/article/10.1007/BF02353786	obtain nominal coverage across all looks	- heuristic approach with no real guarantee or approach how to get the desired coverage		
**28**	A group sequential procedure for all-pairwise comparisons of k treatments based on the range statistic<br><br>Liu (1995)<br>https://doi.org/10.2307/2532995	Repeated simultaneous CIs found by inversion of test statistic.	Normal data in superiority setting with multiple arms when all pairwise questions are of interest.<br><br>CUs have closed form solution with exact (simultaneous) coverage<br><br>Assumes known variance	No	No
**29**	Stopping a clinical trial very early based on unplanned interim analyses: A group sequential approach<br><br>Emerson (1995)<br>https://doi.org/10.2307/2533015	Inversion of test statistics assuming sample mean ordering	Single-arm binary endpoint<br><br>CIs can cope with data dependent unplanned stopping<br><br>Performance of CIs not assessed by simulation	No	No
**30**	Point and interval estimation following a sequential clinical trial<br><br>Todd et al. (1996)	Modification of method of Woodroofe (1992) to construct approximate pivotal quantity based on group sequential test	Applicable to any score statistic based test-statistics<br><br>Point and interval estimates are nicely aligned	Not mentioned in paper, but was implemented in PEST	No

	https://doi.org/10.1093/biomet/83.2.453		Coverage probabilities generally are close to the nominal level but can have undercoverage		
**31**	Sequential monitoring of clinical trials with multiple survival endpoints<br><br>Williams (1996)<br>https://doi.org/10.1002/(SICI)1097-0258(19961115)15:21%3C2341::AID-SIM453%3E3.0.CO;2-N	Three (slightly) different approaches based around inverting linear rank statistics. Distribution of test statistic is approximated using simulations<br><br>1) RCI as in Jennison Turnbull (1989) based on HR<br>2) RCI as in Jennison Turnbull (1989) based on log-HR<br>3) CIs based on Fieller	Multiple TTE endpoints in superiority setting<br><br>Requires asymptotic normality and numeric approximation.<br><br>Coverage is close to nominal<br><br>CIs based on Fieller's methods are significantly wider than RCI based on log-HR, anc can have bounds outside of parameter space	No	Re-analysis of group sequential trial data
**32**	Confidence interval calculation for a sequential clinical trial of binary responses<br><br>Todd and Whitehead (1997)<br>https://doi.org/10.1093/biomet/84.3.737.	Extends methodology of Woodroofe (1992) to binary responses<br><br>Constructs CI for log-odds-ratio of success<br><br>The method is based on constructing a pivotal quantity which is taken as following a standard normal distribution, and then employing traditional probability arguments.	Two-arm sequential trials with binary responses<br><br>Calculating CIs based on Woodroofe's pivot is a substantial improvement over the naive CI in all cases where an improvement is needed.	No	No

**33**	Estimation for an adaptive allocation design<br><br>Rosenberger and Sriram (1997)<br>https://doi.org/10.1016/S0378-3758(96)00109-7	Constructs approximate CIs using asymptotic normal distribution of the MLE	Urn-based RAR designs (using Polya urn) with a random sample size via a stopping rule<br><br>Not evaluated by simulation	No	No
**34**	Thalidomide for the treatment of oral aphthous ulcers in patients with human immunodeficiency virus infection<br><br>Jacobson et al. (1997)<br>https://www.nejm.org/doi/full/10.1056/NEJM199705223362103	Application paper that says its CIs are adjusted for sequential testing, but does not state how or what method was used.	NA	No	Methods applied to real trial
**35**	Approximate confidence intervals after a sequential clinical trial comparing two exponential survival curves with censoring<br><br>Coad and Woodroofe (1997)<br>https://doi.org/10.1016/S0378-3758(96)00204-2	Two-sample extension of Coad and Woodroofe (https://doi.org/10.1093/biomet/83.4.763)	Sequential test comparing two exponential survival curves (with censoring)<br><br>In general, approximation is reasonably accurate	No	No

**36**	Resampling methods for confidence intervals in group sequential trials<br><br>Chuang and Lai (1998)<br>https://doi.org/10.1093/biomet/85.2.317	Uses CIs found in Siegmund (1978) and Tsiatis et al. (1984) and replaces the normal quantiles with quantiles from a resampled distribution (hence a hybrid approach)<br><br>Extension to multiple endpoints also presented	Two-arm group-sequential trials<br><br>Largely distribution free<br><br>CIs only have asymptotic coverage (second-order accurate)<br><br>Computationally intensive	No	No
**37**	Interim analyses using repeated confidence bands<br><br>Hu and Lagakos (1999)<br>https://doi.org/10.1093/biomet/86.3.517	Extends the repeated confidence interval approach to repeated confidence bands for the mean function of a general response process, for example repeated measures of lab markers; failure time endpoints and recurrent event data.	Estimator of mean function that when standardised is asymptotically Gaussian<br><br>Relies on simulation for calculating critical values, which can be computationally intensive	No	Re-analysis of trial data
**38**	Bootstrap methods for adaptive designs<br><br>Rosenberger and Hu (1999)<br>https://doi.org/10.1002/(SICI)1097-0258(19990730)18:14%3C1757::AID-SIM212%3E3.0.CO;2-R	CIs approximated using bootstrap procedure (various approximations using bootstrap replicates proposed), compared with standard CI ignoring adaptions	Focus on RAR using urn models (RPW and Success-driven design)<br><br>Best bootstrap procedure almost always performs better than standard CI in terms of coverage and has similar expected length	No	Re-analysis of RAR trial
**39**	Group sequential procedures for repeated events data with frailty	Extends repeated confidence interval methodology to this trial setting	Group sequential trials with recurrent study endpoints, where the subjects are from a	No	Hypothetical example

	Jiang (1999)<br>https://doi.org/10.1081/BIP-100101183		heterogeneous population. Focus on modelling a Poisson process with frailty.<br><br>RCIs allow the study results to be evaluated flexibly at interim analyses without depending on the rigid stopping criteria<br><br>Requires estimates of certain parameters from pilot data or interim data<br><br>Methods are asymptotic and depend on the large sample approximation		
**40**	Group sequential analyses for the mean function of a repeated measure process<br><br>Hu and Lagakos (1999b)<br>https://doi.org/10.1002/(SICI)1097-0258(19990915/30)18:17/18%3C2287::AID-SIM255%3E3.0.CO;2-D	Extends Hu and Lagakos (1999) above to repeated confidence bands for the mean value of a repeated measures process	Approach does not require much prespecification so may be suited to an area where treatment effects are poorly understood and rapidly evolving<br><br>Flexibility of this approach may make it less efficient than methods with more structure (pre-specification)	No	Hypothetical example
**41**	Hybrid resampling methods for confidence intervals<br><br>Chuang and Lai (2000)<br>https://www3.stat.sinica.edu.tw/statistica/j10n1/10-1.htm	Hybrid methods combining bootstrapping and final/exact CI methods for constructing confidence intervals following a group sequential test<br><br>Called the “hybrid resampling” method because it “hybridizes” the final/exact method (that uses test inversion) with the	General methodological paper with application to group sequential clinical trials<br><br>The final/exact method applies only when there are no nuisance parameters and this assumption is rarely satisfied in practice.<br><br>Bootstrap method can have poor performance when the sampling distribution of the test statistic used varies substantially with the parameter of interest	No	No

		bootstrap method (that uses the observed data to determine the resampling distribution)<br><br>Hybrid resampling method also used in Chuang and Lai (1998)	Hybrid confidence intervals/regions generally have coverage close to the nominal in the simulations presented, whereas the final/exact and bootstrap methods can sometimes have coverage far from the nominal<br><br>Influential observations are troublesome for the bootstrap and remain so for the hybrid resampling method<br><br>Hybrid method is computationally intensive and can be difficult to implement.		
**42**	Computations for group sequential boundaries using the Lan-DeMets spending function method<br><br>Reboussin et al. (2000)<br>https://doi.org/10.1016/S0197-2456(00)00057-X	Describe an interactive Fortran program which performs computations related to the design and analysis of group sequential clinical trials using Lan-DeMets spending functions.<br><br>Uses methodology of Kim and DeMets (1987)	Trial setting as in title<br><br>Computation of CI can be relatively time consuming	Fortran programme	No
**43**	Corrected confidence intervals following a sequential adaptive clinical trial with binary responses<br><br>Coad and Govindarajulu (2000)<br>https://doi.org/10.1016/S0378-3758(00)0	Uses methodology of Woodroofe (1992)<br><br>Constructs an approximately pivotal quantity which is asymptotically standard normal	Sequential trial of two treatments with binary responses<br><br>Can apply to response-adaptive randomisation (e.g. RPW)<br><br>Relies on asymptotic arguments	No	No

	[0129-4]		Corrected CIs can be conservative for certain stopping rules		
**44**	Group sequential designs for cure rate models with early stopping in favour of the null hypothesis<br><br>Bernado and Ibrahim (2000)<br>https://doi.org/10.1002/1097-0258(20001130)19:22%3C3023::AID-SIM638%3E3.0.CO;2-X	Extends repeated confidence intervals for group sequential designs with cure rate model<br><br>Uses asymptotic arguments	Two-arm group sequential designs with cure rate model<br><br>Relies on asymptotic arguments<br><br>No simulation study provided to look at coverage	Method available in EAST software	Hypothetical example
**45**	Flexible interim analyses in clinical trials using multistage adaptive test designs<br><br>Wassmer et al. (2001)<br>https://link.springer.com/article/10.1177/009286150103500410	Repeated confidence intervals for adaptive group sequential designs<br><br>Invert the family of group sequential tests	Adaptive group sequential designs where<br><br>The repeated confidence intervals are valid whatever stopping rules used<br><br>They do not depend on a specific ordering of the sample space or a specific stopping rule<br><br>Strictly conservative	ADDPAL 2001 available from authors on request	Re-analysis of two group sequential trials
**46**	On sample size and inference for two-stage adaptive designs<br><br>Liu and Chi (2001)<br>https://doi.org/10.1111/j.0006-341X.2001.	CIs constructed by inverting overall adjusted p-value at trial termination (assuming monotonicity)	Two-stage adaptive designs using conditional error function<br><br>Not evaluated by simulation	No	No

	00172.x				
**47**	Flexible two-stage designs: An overview<br><br>Bauer et al. (2001)<br>https://www.thieme-connect.de/products/ejournals/abstract/10.1055/s-0038-1634472	Review article<br><br>References repeated confidence intervals, CIs proposed by Liu and Chi (2001) and CIs proposed by Brannath et al. (2002)	Two-stage flexible designs based on combination tests and conditional error functions<br><br>Doesn't assess properties of CIs	No	No
**48**	Sequential confidence regions of generalized linear models with adaptive designs<br><br>Ivan Chang (2001)<br>https://doi.org/10.1016/S0378-3758(00)00200-7	Procedure is based on the maximum quasi-likelihood estimate, without assuming canonical link functions for GLMs.	For regression parameters of a generalised linear model<br><br>Obtain large sample properties of the procedures.	No	No
**49**	Recursive combination tests<br><br>Brannath et al. (2002)<br>https://doi.org/10.1198/016214502753479374	CIs constructed by 1) inverting overall p-value assuming montonicity and 2) repeated confidence intervals following Jennison and Turnbull (1984)	Multi-stage ADs using recursive combination tests<br><br>Monotone CI has close to nominal coverage	No	No

**50**	Sequential tests and estimators after overrunning based on maximum-likelihood ordering<br><br>Hall and Liu (2002)<br>https://doi.org/10.1093/biomet/89.3.699	Constructs CIs by inverting hypothesis test / corresponding p-value	Sequential and group sequential trials with overrunning<br><br>CIs need to be obtained numerically<br><br>Recommends not using conditional inference (conditioning on stopping time) as this contains information about the parameter of interest<br><br>No simulations	No	Hypothetical example
**51**	Confidence intervals following group sequential tests in clinical trials with multivariate observations<br><br>Lee et al. (2002)<br>https://doi.org/10.1080/00949650212386	Proposes final/exact confidence intervals in a group sequential trial with repeated measures<br><br>Extends methodology of Kims and DeMets (1987)	Trial setting as in title<br><br>Simulation compares proposed CI with naive/standard CI.<br><br>Both the final/exact confidence interval and the naive confidence interval underestimate the desired coverage probabilities on the average, but the final/exact confidence interval gives the closer coverage probabilities. The naive confidence interval underestimates the desired coverage probability especially when the within-subject variation is large compared to the between-subject variation.	No	Hypothetical example
**52**	A unified theory of two-stage adaptive designs<br><br>Liu et al. (2002)<br>https://doi.org/10.1198/016214502388618	Theoretical result about the existence of pivotal quantities which can be used to construct CIs	General two-stage ADs	No	No

	852				
**53**	Conditional inference following group sequential testing<br><br>Ohman Strickland and Casella (2003)<br>https://doi.org/10.1002/bimj.200390029	Conditional confidence intervals for group sequential designs (conditional on the stopping time and the direction of the sample mean away from the hypothesized null value)<br><br>Conditional CIs are found by imposing a stochastic ordering of the sample space (e.g. stagewise) and then pivoting the CDF.	Conditional intervals are extremely wide when values of s (the test statistic when the trial has ended) are close to the critical values<br><br>When null hypothesis has been rejected and value of s is close to the boundary the null value will be included in the conditional interval<br><br>But make more intuitive sense for unlikely results, i.e. make better use of information which is known at the time the experiment is stopped when s is far from the boundary<br><br>Arbitrariness of the ordering of the sample space is removed by conditioning.<br><br>Conditional coverage of usual adjusted CIs (both final/exact and repeated) can be extremely variable, ranging from 0 to 1	No<br><br>R code used but not provided	No
**54**	Improved repeated confidence bounds in trials with a maximal goal<br><br>Brannath et al. (2003)<br>https://doi.org/10.1002/bimj.200390014	Proposes an improved repeated CI for group sequential designs where it is known in advance that the trial will stop if the test statistic is larger than a certain value<br><br>Extends the methodology of Lehmacher and Wassmer	Focuses on flexible two-stage group sequential designs<br><br>The resulting one-sided intervals are uniformly smaller than the repeated confidence intervals when stopping the trial at an interim analysis while reaching the maximal goal. In this case repeated confidence bounds are particularly conservative. In the other cases the RCI is	No	No

		(1999) and Jennison and Turnbull (1989)	left unmodified, and so their flexibility is preserved.		
**55**	Practical midcourse sample size modification in clinical trials<br><br>Proschan et al. (2003)<br>https://doi.org/10.1016/S0197-2456(02)00240-4	CIs constructed by inverting the normal hypothesis test	Sample size re-estimation (taking place at any pre-planned point during the trial)<br><br>CIs not evaluated	No	Hypothetical example
**56**	Interval estimation in adaptive allocations using point estimates<br><br>Bandyopadhyay and Biswas (2003)<br>https://doi.org/10.1177/0008068320030103	Quite general approach to constructing a CI for a (continuously monitored) response adaptive rule - consider the (exact) probability of observing all sequences of allocations. Build a conditional CI for each possible allocation and weight them.	Consider randomized play-the-winner and two other response adaptive rules.<br><br>Continuous responses<br><br>Properties of CIs not evaluated by simulation	No	Hypothetical example
**57**	Estimation following group-sequential response-adaptive clinical trials<br><br>Morgan (2003)<br>https://doi.org/10.1016/S0197-2456(03)00062-X	It derives bias-corrected confidence intervals for group-sequential response-adaptive designs proposed in Chapter 17 of the SGD book by Jennison and Turnbull. It uses a Taylor series expansion to approximate the	Response-adaptive group-sequential 2-arm multi-stage design with continuous outcomes.<br><br>The proposed corrected-CI provides much better coverage probabilities, and differed markedly with that of the naive CI. The proposed method works better in 95% CIs.	No	No

		expected value of the test-statistics	Simulation results show that the naıve coverage probabilities tend to be too low for effect sizes close to half of the target values under the alternative hypothesis and too high for extreme effect sizes.		
**58**	Data-driven analysis strategies for proportion studies in adaptive group sequential test designs<br><br>Wassmer (2003)<br>https://doi.org/10.1081/BIP-120024196	The article applies the standard techniques for adaptive group sequential designs to 2-arm multi-stage trials with a binary outcome. It provides the sample-size formula for sequential testing of risk difference and risk ratios between the research arm and the control group. The proposed testing procedure is based on the weighted combination of the normal scores of the separate stages where the weights are prefixed prior to the start of the study.<br>The article proposes an iterative procedure to calculate repeated confidence intervals.	No analytical or closed-form formula is given.<br><br>The repeated confidence intervals are determined by finding an effect size that do not lead to a rejection of the corresponding null hypothesis when using the p-value of the test statistic.<br><br>The article does not provide empirical evidence as how the proposed approach compares with the uncorrected confidence intervals.	No, but the authors suggest that all the numerical calculations can be done in ADDPLAN program	No
**59**	Improved confidence intervals for a binomial parameter following a group sequential phase II clinical trial<br><br>Chang (2004)	The article proposes to define condence intervals that are formed based on a “refined” (or more restricted) sample space. They propose this approach in the context of phase II setting	Group sequential design, and extend the approach for the ordering of the sample space by Jennison and Turnbull (JT). The proposed approach provides shorter intervals with much better/improved coverage probability (i.e. closer to the	Yes in SAS	No

	https://doi.org/10.1002/sim.1878	with a single experimental arm.	nominal values) than that of the JT approach.		
**60**	Estimation of a parameter and its exact confidence interval following sequential sample size reestimation trials<br><br>Cheng and Shen (2004)<br>https://doi.org/10.1111/j.0006-341X.2004.00246.x	Proposes an approach to calculate CIs for a group sequential design with unblinded sample size re-estimation. The authors derived the confidence interval based on the distribution of a constructed pivot function, which is a function of the final-stage test statistic and the parameter of interest	The method allows for designs with only futility stopping rule which has to be pre-specified. The analytical derivations were done asymptotically (only the large sample properties studied) under the assumption of continuous outcomes.	No	No
**61**	Adaptive design and estimation in randomized clinical trials with correlated observations<br><br>Yin and Shen (2005)<br>https://doi.org/10.1111/j.1541-0420.2005.00333.x	The article presents a flexible design and inference procedure for correlated observations in clustered randomised trials that use the GEE as an analysis method. The proposed method is applicable in a wide variety of situations including discrete and continuous correlated outcome data using regression models. In this approach the sample size is updated (or re-estimated) given the observed trend in the accumulated data	The design only allows for interim futility based on conditional power.<br><br>CIs based on asymptotic properties<br><br>The 95% confidence interval coverage rates are slightly lower than the nominal level.	No	Hypothetical example

**62**	Drop-the-Losers Design: Normal Case<br><br>Sampson et al. (2005)<br>https://doi.org/10.1002/bimj.200410119	Develops uniformly most powerful conditional CIs based on inverting hypothesis tests (using CDF of montone likelihood ratio). Conditional on ordering of observed treatment means.	Drop-the-loser trial designs with normally-distributed endpoints<br><br>Conditional CIs in example have shorter length than Bonferroni and just using second stage data, but is larger than naive CI (whose coverage is less than 95%)	Yes but links are now dead	No
**63**	Point estimates and confidence regions for sequential trials involving selection<br><br>Stallard and Todd (2005)<br>https://doi.org/10.1016/j.jspi.2004.05.006	Constructs confidence region based on inverting p-value function (based on ordering the sample space)<br><br>Confidence interval for selected treatment mean also considered by assuming all other treatment means are equal to 0 or their observed mean	Sequential trial where treatment selection occurs at the first of two or more interim analyses<br><br>Standard/naive confidence intervals (based on an assumption of normality) are not accurate, whereas the confidence regions based on inversion of the p-value function do have accurate coverage<br><br>In general, it appears that the CI assuming all other treatment means are equal to 0 gives more accurate coverage than the standard CI<br><br>The confidence region is necessarily unbounded<br><br>Confidence regions based on inversion of the p-value function are not necessarily consistent with the estimation method used.	No	Hypothetical example

			This could lead to confidence regions that do not include the point estimate.		
**64**	Testing and estimation in flexible group sequential designs with adaptive treatment selection<br><br>Posch et al. (2005)<br>https://doi.org/10.1002/sim.2389	The article reviews adaptive treatment selection based on combination tests and proposed overall adjusted p-values and simultaneous confidence interval.	The resulting confidence intervals may be inconsistent with the test decision (the confidence interval may not exclude the parameter values which were rejected in the original multiple test procedure).<br><br>Also, the article focuses more on testing and point estimation when selecting/dropping research arms. Only one small section is dedicated to CIs.	No	No
**65**	Conditional and unconditional confidence intervals following a group sequential test<br><br>Fan and DeMets (2006)<br>https://doi.org/10.1080/10543400500406595	It proposes a two-step restricted conditional confidence interval (RCCI), which captures most of the information contained in both the stopping time and the test statistic. The approach improves on the properties of conditional confidence interval methods.	The proposed method improves the conditional coverage probability considerably from the final/exact unconditional CI's but also is free of the major undesirable properties displayed by the pure conditional CI.<br><br>The article also explores the differences between the conditional and unconditional CI's and their respective strengths.<br><br>The RCCI can be possibly an empty set if the two intersecting intervals do not overlap	No	No
**66**	Planning and Analyzing Adaptive Group Sequential Survival Trials	The article proposes a flexible adaptive design for trials with time to-event outcomes, which allows for the interim sample	The article proposes CI based on the well-known (stagewise) ordering of the sample space. This results in conservative confidence intervals.	Yes, in ADDPLAN	Hypothetical example

	Wassmer (2006)<br>https://doi.org/10.1002/bimj.200510190	size reestimation. The method allows for data-driven changes in design, under control of the Type I error rate. It also proposes estimation methods both for confidence interval and point estimation.			
**67**	Repeated Confidence Intervals in Self-Designing Clinical Trials and Switching between Noninferiority and Superiority<br><br>Hartung and Knapp (2006)<br>https://doi.org/10.1002/bimj.200510213	It proposes repeated confidence intervals for adaptive group sequential designs which allow for sample size re-estimation. The design also allows the change of testing procedure from superiority to non-inferiority and vice versa. The approach is based on P-value combination methods.	The proposed method is developed for sequential designs with continuous outcomes. The final stage confidence intervals are over-conservative	No	Hypothetical example
**68**	Adaptive vs. group sequential self-designing trials<br><br>Mehrotra and Fan (2006)<br>https://doi.org/10.1002/bimj.200610259	Discussion of above paper<br>Point to practical issues of above linked to the inefficiency of the AD considered.<br>Also point to the fact that when switching hypotheses you could intersect later CIs with earlier CIs.	As above.	No	No
**69**	Rejoinder	Rejoinder to above	As above.	No	No

	Hartung and Knapp (2006)<br>https://doi.org/10.1002/bimj.200610259	Point out some differences identified by Mehrotra and Fan are due to the use of a different p value combination function.			
**70**	Estimation in flexible two-stage designs<br><br>Brannath et al. (2006)<br>https://doi.org/10.1002/sim.2258	Flexible CIs constructed by 1) inverting hypothesis tests (for trials without early stopping) and 2) extending the repeated confidence interval (RCI) approach of Jennison and Turnbull to flexible designs<br><br>Proposes enlarging these adjusted confidence so that they always contain the classical CI (and hence the MLE)<br><br>Also constructs flexible CI based on the sufficient test statistic (centred on MLE): use likelihood ratio test static and adjust the critical boundary for potential adaptations	Two-stage flexible adaptive designs, with a focus on designs using weighted z-score method (combining stagewise z-scores) and sample size reassessment rules<br><br>Flexible CIs for trials without early stopping are consistent with hypothesis test decision with exact coverage probability<br><br>Flexible RCIs have conservative coverage probability<br><br>Flexible CIs do not always contain the classical CI or the MLE, and do not use the sufficient test statistics<br><br>Flexible CIs based on the sufficient test statistic are large compared to classical CI and larger than flexible CI (apart from extreme adaptations) - is a worst case adjustment of the critical boundary over all possible sample size reassessment rules<br><br>Relative length of flexible RCIs compared with flexible CI based on sufficient test statistic depends on “the strength and frequency of the sample size reassessments: if sample size adaptations	No	Re-analysis of trial data

			are seldom and moderate then RCI will be shorter”		
**71**	Confidence intervals in group sequential trials with random group sizes and applications to survival analysis<br><br>Lai and Li (2006)<br>https://doi.org/10.1093/biomet/93.3.641	Extends the hybrid method of Chuang & Lai (2000) of constructing CIs to account for random group sizes in a group sequential trial<br><br>Application to time-sequential tests with the Cox model	CIs only has asymptotic coverage (second-order accurate)<br><br>Simulations show proposed method has coverage quite close to the nominal for finite sample sizes (within 1%)	No	No
**72**	Adaptive design: Estimation and inference with censored data in a semiparametric model<br><br>Shen and Cheng (2007)<br>https://doi.org/10.1093/biostatistics/kxl011	The article proposes a new adaptive (GSD) trials with time-to-event outcomes. It derives an analytical formula for the bias-adjusted estimator of the treatment effect. The proposed approach also allows for covariate adjustment. It derives a confidence interval based on the asymptotic properties of the proposed bias-adjusted estimators.	The design only allows for interim stopping for futility.<br><br>The proposed (asymptotic) confidence interval provides accurate coverage probability across all simulation scenarios with or without adjusting for covariates.	Yes	No
**73**	Flexible design of two-stage adaptive procedures for phase III clinical trials<br><br>Koyama (2007)	CIs constructed by inverting p-values	Two-stage flexible adaptive designs with normal responses and sample size reassessment<br><br>Properties of CIs not assessed	No	No

	https://doi.org/10.1016/j.cct.2007.01.007				
**74**	Repeated confidence intervals for adaptive group sequential trials<br><br>Mehta et al. (2007)<br>https://doi.org/10.1002/sim.3062	The article proposes a method for computing conservative confidence intervals for a group sequential trial design. The method uses the procedure developed by Muller and Schafer (which is based on the conditional error rate principle) for adaptive group sequential hypothesis testing.	The proposed procedure is conservative. The simulation results suggest that it provides higher coverage than the nominal values. Limitations are as follows: It only allows for one-sided settings and one-sided tests; it can not be applied to equivalence trials.	Yes	No
**75**	Interim treatment selection using the normal approximation approach in clinical trials<br><br>Shun et al. (2007)<br>https://doi.org/10.1002/sim.2990	Approximate conditional CI found based on normal approximation of test statistics and estimating parameters using interim data. Conditional on treatment selected to stage 2.	Two-stage drop-the-loser design with two treatment groups (+ control) and normal responses<br><br>Properties of proposed CI not evaluated	No	No
**76**	Point and interval estimation of primary and secondary parameters in a two-stage adaptive clinical trial<br><br>Liu et al. (2007)<br>https://doi.org/10.1080/10543400701697125	CIs for primary parameter using methods of Woodroofe (1992) and extended by Whitehead et al. (2000), Todd and Whitehead (1997), Todd et al. (1996): construct an approximately normal distributed pivotal quantity	Two-stage design with sample size re-estimation with both primary and secondary endpoints<br><br>Coverage probability for proposed CIs are very close to the nominal in the simulation studies	No	No

		CIs for secondary parameter also found by constructing an approximately normal distributed pivotal quantity			
**77**	A note on repeated p-values for group sequential designs<br><br>Posch et al. (2008)<br>https://doi.org/10.1093/biomet/asm080	This article is about the impact of the ordering of the sample-space on both the estimated p-values and corresponding confidence intervals. It shows that the commonly used repeated confidence intervals (and p-values) are not ordering-consistent. It proposed alternative repeated p-values and (confidence intervals) which are ordering consistent that are valid even when deviating from the pre-specified stopping rules.	This article is on a rather related topic of the ordering of the sample space. It does not provide a new method on the calculation of CI. Therefore, no limitations is mentioned in this regard.	No	No
**78**	Accounting for Interim Safety Monitoring of an Adverse Event Upon Termination of a Clinical Trial<br><br>Dallas (2008)<br>https://doi.org/10.1080/10543400802071	This article applies final/exact CI approach for GSDs to account for interim safety monitoring	As anticipated, the approach provides exact coverage probabilities. It also performs better than the conventional approaches based on the asymptotics in the simulation studies. The method is only developed for binary outcomes.	No	No

	311				
**79**	Point and interval estimation of accuracies of a binary medical diagnostic test following group sequential testing<br><br>Shu et al. (2008)<br>https://doi.org/10.1098/rsta.2008.0041	Approximate CIs (of sensitivity and specificity) upon termination are constructed using Woodroofe's pivot method (1992). These CIs are based on different plug-in estimators such as maximum likelihood estimators and Rao-Blackwell unbiased estimators	Two-arm group sequential involving a diagnostic accuracy study (i.e., joint hypotheses concerning sensitivity and specificity) of a binary diagnostic test<br>They seem to yield less coverage probabilities than the nominal level, especially when estimates are closer to the boundaries although they are (slightly) better than naïve CIs (this is based on simulation results assuming small sample sizes and two-stage design).	No	No
**80**	Proper inference from Simon's two-stage designs<br><br>Koyama and Chen (2008)<br>https://doi.org/10.1002/sim.3123	The CIs calculated by inverting hypothesis test where a p-value is computed based on a conditional power function that does through the conditional p-value	Simon's two stage design<br><br>Incorporates both planned and actual sample sizes (when change in sample sizes is non-informative)<br><br>Authors did not explore the performance of proposed CI	Yes, but link seems to be broken	No
**81**	On adaptive extensions of group sequential trials for clinical investigations<br><br>Liu and Anderson (2008)<br>https://doi.org/10.1198/016214508000000986	Sequential upper and low confidence bounds (CIs) at each interim analysis centred around the (median) unbiased estimator are calculated based on ordering of the sample space and inverting group sequential tests (as described by Jennison and Turnbull, 2000)	Group sequential where a trial is allowed to continue to collect additional data (e.g., secondary outcomes, safety, pipeline primary outcome responses) after a stopping boundary is crossed based on the primary outcome.	No	No

		with minor adjustments. These CIs are sequential intersections of the repeated confidence intervals of Jennison and Turnbull.	Actual trial stopping time is based on the totality of accumulating evidence.<br><br>Allows for adaptation to the sample size.<br><br>Use of variance-spending approach that allows incorporation of all trial data with some weights.<br><br>**Limitations**<br><br>Assumes homogeneity of treatment effect across interim analyses (*which is not unique to this method really*)		
**82**	Drop-the-losers design: Binomial case<br><br>Sill and Sampson (2009)<br>https://doi.org/10.1016/j.csda.2008.07.031	Conditional CIs found by inverting uniformly most powerful conditionally unbiased hypothesis tests. Conditional on observed ranking of treatment means.	Drop-the-loser designs with binary responses<br><br>CIs are exact theoretically but due to data discreteness can deviate in realised designs	No	No
**83**	Conditional estimation of sensitivity and specificity from a phase 2 biomarker study allowing Early termination for futility<br><br>Pepe et al. (2009)<br>https://doi.org/10.1002/sim.3506	Proposes two resampling/bootstrap methods for constructing CIs:<br><br>1) 'Parametric bootstrap': use the estimated UMVCUE to simulate N studies, calculate	Two-stage group sequential design with a binary outcome.<br><br>The parametric bootstrap allows unconditional estimation and confidence interval calculations, if desired. With the nonparametric bootstrap, only conditional estimation and confidence interval calculation are possible, but it can be	No	No

		UMVCUE (for those replicates where the continuation criterion is satisfied) and use empirical distribution<br><br>2) ‘Non-parametric’ bootstrap: resample responses with replacement, then calculate UMVCUE (for those replicates where the continuation criterion is satisfied) and use empirical distribution	applied more generally (without distributional assumptions)<br><br>Coverage is reasonably close to the nominal 95 per cent level for both bootstrap methods, but somewhat lower for the parametric bootstrap than for the nonparametric bootstrap. Correspondingly, the standard deviation tends to be slightly underestimated with the parametric methods, but overestimated with the nonparametric bootstrap		
**84**	Statistical monitoring of clinical trials with multivariate response and/or multiple arms: A flexible approach<br><br>Zhao et al. (2009)<br>https://doi.org/10.1093/biostatistics/kxn037	Multivariate repeated confidence regions/bands for trial monitoring (i.e., at each interim analysis) while retaining desired<br><br>It is a generalisation of repeated confidence interval for a single parameter by Jennison and Turnbull, 1989<br><br>Either a spending function (like Lan and DeMets) is applied to determine the confidence regions or confidence regions determine the spending function	Flexible monitoring approach where specific aspects that influence early stopping (e.g., decision rules) cannot be prespecified and decision metrics can change as the trial progresses.<br>Covers adaptive trials with multi-arm or/and where early stopping is based on multivariate/multiple outcomes (e.g., efficacy and safety for benefit/risk balance)<br><br>Decision to stop is based on totality of evidence (scope defined by investigators and can change over time) and confidence bands that retains desired frequentist characteristics.<br><br>Flexible and doesn’t require model specification (e.g., of the mean function)	No	Hypothetical example

			thus protecting against model misspecification<br><br>Reasonable approach to benefit/risk monitoring<br><br>Its efficiency needs to be explored further under different scenarios (e.g., target function of the mean)		
**85**	Adaptive designs for confirmatory clinical trials<br><br>Bretz et al. (2009)<br>https://doi.org/10.1002/sim.3538	Tutorial paper, reviews concept of Repeated Confidence Intervals (Jennison and Turnbull, 1984), Final/Exact Confidence Intervals (Tsiatis et al. 1984) and Simultaneous Confidence Intervals (Posch et al. 2005)	Repeated confidence intervals are typically strictly conservative but give valid confidence intervals even if one does not adhere to the pre-specified stopping rules. The final/exact confidence bounds in contrast are exact but can only be computed at stages where a stopping boundary has been crossed or in the final analysis.<br><br>The RCIs may not contain the MLE.<br><br>For simultaneous confidence intervals, enlarging the confidence region to a rectangle the resulting confidence intervals may be incompatible with the test decision (the confidence interval may not exclude the parameter values that were rejected in the original multiple test procedure).	No	No
**86**	Confirmatory adaptive designs with Bayesian decision tools for a targeted therapy in oncology	Constructs one-sided repeated confidence intervals for the Cov-regression parameter. Also constructs simultaneous	Seamless phase II/III trial with population selection at the first interim analysis (either select sub-population or continue with the	No	No

	Brannath et al. (2009)<br>https://doi.org/10.1002/sim.3559	confidence intervals.	full population) and the possibility to stop early at the second interim analysis.<br><br>RCIs in general exclude 0 when rejecting hypothesis, but may also exclude 0 while the adaptive closed test accepts the respective null hypothesis, as they do not account for the multiplicity in estimating two parameters.<br><br>Multiplicity adjusted confidence intervals (simultaneous confidence intervals) are more conservative and not always consistent (it may happen that the lower confidence bound for the treatment effect in population P is negative; however, the closed test rejects H_P).<br><br>“Confidence intervals that account for the multiplicity and are consistent with the flexible closed test are currently not available.”		
**87**	Exact confidence bounds following adaptive group sequential tests<br><br>Brannath et al. (2009)<br>https://doi.org/10.1111/j.1541-0420.2008.01101.x	Final stagewise adjusted confidence intervals (SWACIs) that provide exact coverage and constructed using stagewise ordering of the sample space.<br><br>Method is based on computing the conditional error probability at the stage when adaptive changes are made and preserving this conditional error probability for the remainder of the trial given the changes	Two-arm GSDs with additional adaptations such as changes to sample size, timing and frequency of interim analyses, and spending functions (referred to as adaptive group sequential designs).<br><br>Focuses on constructing one-sided intervals in the context efficacy early stopping, but extensions to two-sided intervals and futility early stopping are discussed<br>SWACIs are consistent in the sense that if the null hypothesis is rejected then the	Implemented in East-5 (2007) package (suspect it is still implemented in updated versions)	Hypothetical example

		made. This enables decision boundaries after adaptive changes to be adjusted correctly.	confidence interval will exclude the targeted effect size. Related point estimates are median unbiased.<br><br>Applicable regardless of whether additional adaptations (referred above) are made or not. If no additional adaptations are not made, stagewise adjusted confidence intervals applicable for standard GSDs may also be used (e.g., as described by Kim and DeMets and Kim)		
**88**	Tests and confidence intervals for secondary endpoints in sequential clinical trials<br><br>Lai et al. (2009)<br><br>https://doi.org/10.1093/biomet/asp063	It is based on a composite null hypothesis structure on the secondary endpoint, ordering of the test statistics sample space (until the point of trial stopping), and use of hybrid sampling to construct confidence intervals for secondary endpoints. The hybrid sampling method crossbreeds the final/exact method (that uses test inversion) and bootstrap method (uses observed data to determine the sampling distribution of parameters of interest). This approach is well described by Chuang and Lai, 2000.	CIs for secondary endpoints following a sequential trial (fully sequential and group sequential) whose stopping rule depends on the primary endpoint. The underlying common distribution of outcomes (primary and secondary endpoints) can be parametric, semi-parametric, or non-parametric.<br><br>Generally applicable for parametric, semi-parametric, or nonparametric common distributions<br><br>Compares the performance of naïve, bootstrap, and hybrid sampling methods with respect to coverage errors and coverage probabilities by siulation. Concluded that hybrid resampling CIs are “considerably more accurate and reliable”.<br><br>Coverage probability of CIs is not exact (still residual error).<br><br>Seems to be computationally intensive	No	No

**89**	Adaptive group sequential confidence intervals for the ratio of normal means<br><br>Hartung and Knapp (2010)<br>https://link.springer.com/article/10.1007/s13571-010-0005-5	Derives nested multistage CIs based on accumulated data at each interim analysis by intersecting the lower and upper one-sided CIs (across relevant stages).	Two-arm group sequential with normally distributed outcomes with ratio of means as treatment effect of interest<br><br>Allows adaptation to sample size<br><br>Applicable in superiority or non-inferiority trials<br><br>Allows switching of hypothesis (e.g., non-inferiority to superiority) at each interim analysis<br><br>Data across stages are combined using inverse normal combination method/function<br><br>In rare cases, it can produce empty confidence intervals and the probability of this happening is bounded by 2α (as described by Hartung and Knapp, 2010)<br><br>Did not explore the performance of proposed CIs through simulation	No	Re-analysis of group sequential trial data
**90**	Interval estimation for drop-the-losers designs	Proposes CI based on inverting hypothesis test under least favourable configuration for the coverage probability of the	Two-stage drop-the-loser trial with normally-distributed outcomes<br><br>Compares proposed CIs with the one proposed by Sampson and Sill (2005)	No	Hypothetical example

	Wu et al. (2010)<br>https://doi.org/10.1093/biomet/asq003	interval estimator. CIs proposed for both a mean combination decision rule and a p-value combination decision rule	All CIs are consistent with their corresponding tests (i.e. a test claims superiority of a treatment if and only if the corresponding lower confidence limit is greater than 0).<br><br>Power of proposed CIs usually higher than the Sampson and Sill test.<br><br>Compared to the mean combination test, the p-value combination method lost some efficiency.		
**91**	Adaptive confidence intervals of desired length and power for normal means<br><br>Hartung and Knapp (2010)<br>https://doi.org/10.1016/j.jspi.2010.04.054	Presented multi-stage confidence intervals constructed based on computing stagewise independent test statistics. The cumulative test statistics at each stage are then calculated using an inverse-normal combination test function. Lower confidence sets across stages are defined such that if the trial stops early, the coverage will be larger than expected and exact (as desired) if it stops at the planned final stage. The maximum and minimum of the confidence sets corresponds to the upper and lower confidence interval limits, respectively.	Confidence interval for a one sample mean of normally distributed outcome so it does not have immediate application in comparative multi-stage trials (although the idea may be extended)<br>Properties of the constructed intervals have not been fully studied under different scenarios.<br><br>In some cases, it can result in empty confidence interval set (i.e., the upper limit being smaller than the lower limit)<br><br>Seems to produce confidence intervals with desired coverage only when the trial progresses to the planned end with maximum sample size. Otherwise, coverage will be larger than expected	No	Re-analysis of trial data

**92**	Likelihood inference for a two-stage design with treatment selection<br><br>Bebu et al. (2010)<br>https://doi.org/10.1002/bimj.200900170	A conditional likelihood-based approach is proposed to construct confidence intervals for the parameters of interest in a two-stage design with treatment selection after the first stage. Both a Wald confidence interval and a confidence interval based on inverting the likelihood ratio test are proposed.	Two-arm trial with treatment selection (treatment with largest sample mean selected)<br><br>Compares proposed CIs with those proposed by Bowden and Glimm (2008) and Sampson and Sill (2005)<br><br>The two proposed confidence intervals have coverage probabilities very close to the nominal level,while having shorter average lengths than the Bowden and Glim confidence interval. Furthermore, they are more stable in controlling the coverage probability under increasing variability.<br><br>Bowden and Glimm CI often is conservative but can also have coverage far below the target	No	No
**93**	Estimation and Confidence Intervals for Two-Stage Sample-Size-Flexible Design with LSW Likelihood Approach<br><br>Wang et al. (2010)<br>https://link.springer.com/article/10.1007/s12561-010-9023-0	CIs derived by inverting hypothesis test (p-values)	Sample-size adaptive design of Li et al. (Biometrics, 2005) based on conditional power considerations<br><br>Proposed CI gives the correct coverage probability for all situations considered	No	Hypothetical example

**94**	Statistical inference in adaptive group sequential trials with the standardized mean difference as effect size<br><br>Hartung and Knapp (2011)<br>https://doi.org/10.1080/07474946.2011.539926	Derives nested multistage CIs based on accumulated data at each interim analysis by intersecting the lower and upper one-sided CIs (across relevant stages). Can be viewed as some form of repeated CIs	Two-arm group sequential with normally distributed outcomes with difference of means as treatment effect of interest<br><br>Allows adaptation to sample size<br><br>Applicable in superiority or non-inferiority trials and allows switching of hypothesis (e.g., non-inferiority to superiority) at each interim analysis<br><br>Data across stages are combined using inverse normal combination method/function<br><br>Seems to produce very good and improved coverage compared to naïve CIs<br><br>In rare cases, it can produce empty confidence intervals (as described by Hartung and Knapp, 2010)	Real example for illustration and a simulation study	No
**95**	Interval estimation for response-adaptive clinical trials<br><br>Tolusso and Wang (2011)<br>https://doi.org/10.1016/j.csda.2010.06.016	Two methods used (based on asymptotic normality of the MLE):<br><br>1) Profile likelihood method of Wei et al. (1990)<br><br>2) Jeffrey-Perks Method: inverts	RAR, namely the randomised play-the-winner rule<br><br>For small values of treatment difference (say less than 0.5 in absolute value) the Jeffreys–Perks method is superior since it results in shorter intervals, while at the same time being conservative. When the treatment difference is large (say greater than 0.5 in absolute	No	Re-analysis of RAR trial

		a test statistic based on Bayes estimator of sum of success probabilities	value) the profile based method is superior since it is shorter and has an actual confidence level closer to nominal.<br><br>Jeffrey-Perks method is simpler computationally (exact formulae) rather than numerical methods for profile likelihood		
**96**	Repeated confidence intervals under fractional brownian motion in long-term clinical trials<br><br>Zhang (2011)<br>https://doi.org/10.1080/03610918.2011.563008	Use fractional Brownian motion (a class of stochastic process) accounting for the Hurst parameter estimated from the trial to compute repeated confidence intervals (RCIs) at each interim analysis (stage) regardless of the stopping decision. It is an extension or generalisation of the standard Brownian motion (BM) process. Unlike in a standard BM process, data increments (from stagewise aggregated data) do not need to be independent.<br><br>The width of fractional Brownian motion RCIs for given values of the Hurst parameter is compared to CIs without any adjustment and to those based on a standard Brownian motion.	RCIs when data from each stage of the trial are not independent (i.e., stagewise interim statistics not having independent increments) which can happen in survival and long-term longitudinal outcomes. RCIs for two-sided tests are presented in two-arm trials (but mentioned that approach can be extended).<br><br>Accounts for at least the dependent in stagewise data increments in the covariance structure and provides a generalised framework through the Hurst parameter (i.e., it reduces to the standard BM process if there is no dependence in data increments).<br><br>The performance of the RCIs depends on other factors such as the stopping rule used and timing of interim analyses. For example, they found that if OBF spending function is used, then the resultant RCI will be wider than expected (conservative).	No	Hypothetical example

**97**	Interval estimation in two-stage, drop-the-losers clinical trials with flexible treatment selection<br><br>Neal et al. (2011)<br>https://doi.org/10.1002/sim.4308	Constructs a lower confidence limit for the mean difference between the selected treatment and the control<br><br>Extends the results of Wu et al. (2010) to flexible selection rules, again deriving the least favourable configuration	Two-stage, drop-the-losers clinical trial with normally-distributed endpoints in which the 'best' treatment may be chosen after the first stage for reasons other than the highest efficacy in the primary clinical endpoint (e.g. first-stage safety data, early endpoint efficacy data, a combination of safety and efficacy data or any other prespecified selection rule)<br><br>Proposed methods have correct coverage probability	No	No
**98**	Conditional estimation after a two-stage diagnostic biomarker study that allows early termination for futility<br><br>Koopmeiners et al. (2012)<br>https://doi.org/10.1002/sim.4430	Calculates conditional CIs for ROC or PPV (or NPV) curve (conditional on reaching full enrolment) based on asymptotic normality and following the approach by Ohman Strickland & Casella (2003, Biom J).<br><br>Unadjusted estimates of nuisance parameters are plugged into variance formulas to calculate conditional CIs.	Two-stage group-sequential diagnostic biomarker study allowing early stopping for futility<br><br>Uniformly most accurate (1-α)-level conditional CIs<br><br>Conditional CIs wider than 'unadjusted' CIs	No	No
**99**	On efficient two-stage adaptive designs for clinical trials with sample size adjustment	Inverts sequential test for adaptive GSDs proposed by Liu & Anderson (2008, JASA) to obtain sequential confidence	Two-stage adaptive design with sample size adjustment (derived from a 'pseudo'-GSD using cumulative conditional power)	No	No

	Liu et al. (2012)<br>https://doi.org/10.1080/10543406.2012.678226	bounds for the interim analysis.<br><br>For the final analysis, the lower sequential bound is calculated as the maximum of the lower bound for the interim analysis and the repeated lower bound; similarly, the upper sequential bound is calculated as the minimum of the upper bound for the interim analysis and the repeated upper bound.	Properties of proposed CIs not evaluated by simulation		
**100**	Stopping a trial early-and then what?<br><br>Wittes (2012)<br>https://doi.org/10.1177/1740774512454600	CIs discussed are calculated as follows:<br><br>1) Start with defining the p-value function based on stagewise ordering,<br><br>2) Use numerical integration or grid search to find the lower and upper confidence limit such that the p-value function equals to α/2 and (1- α/2) for a two-sided test or α and (1- α) for a one-sided test, respectively.<br><br>Stagewise ordering is as described by Kim and DeMets, 1987 and Kim 1989	Discusses bias in estimation (point estimates, CIs and p-value) following early stopping in group sequential trials with illustration using case studies.<br><br>It does not depend on future unobserved data (i.e., calculated based on observed data up to the stage of stopping)<br><br>In certain extreme situations, stagewise based confidence intervals may not contain the (uncorrected) maximum likelihood estimate, which may be confusing to some. However, this may be understandable as the MLE produces a biased estimate of the treatment effect.	They reference Landem program by Reboussin et al (https://pubmed.ncbi.nlm.nih.gov/10822118/)<br><br>https://biostat.wiscweb.wisc.edu/resources/software/programs-for-computing-group-sequential-boundaries-using-the-lan-demets-method/	Re-analysis of group sequential trial data

**101**	Group sequential tests for delayed responses (with discussion)<br><br>Hampson and Jennison (2013)<br>https://doi.org/10.1111/j.1467-9868.2012.01030.x	CIs at the point of early stopping using stagewise ordering of the sample size. Incorporates data accrued after early termination. CIs obtained by inverting a family of hypothesis tests.	Two-arm group sequential trials that anticipates pipeline participants (i.e., outcome data not yet matured/gathered at the point of interim analysis/early stopping)<br><br>Inference incorporates data accrued after early termination of the trial.<br><br>One-sided hypothesis testing but can be extended to two-sided tests<br><br>Monotonicity of ‘p-value’ to construct confidence limits not guaranteed (but conservative CI still can be defined in this case)<br><br>Properties of CIs not evaluated by simulation	No	Hypothetical examples
**102**	Considerations on covariates and endpoints in multi-arm multi-stage clinical trials selecting all promising treatments<br><br>Jaki and Magirr (2013)<br>https://doi.org/10.1002/sim.5669	Constructs multi-dimensional analogues of repeated CIs for GSDs by inverting the test procedure, yielding CIs for each comparison at each stage of the trial.<br><br>Also derives CIs based on stagewise ordering for the final stage of the trial.	Multi-arm multi-stage designs where all promising treatment arms are taken forward after each interim analysis<br><br>Simultaneous lower bounds can be found at each interim analysis<br><br>No longer valid if a step-down test is used to account for individual null hypotheses being rejected early<br><br>Properties of CIs not evaluated by simulaiton	No	No
**103**	Adaptive designs for noninferiority trials	Symmetric stagewise adjusted CIs with exact coverage around	Two-arm adaptive group sequential trials (i.e., allows additional adaptations such as	Cites EAST 5.4 for multistage designs	No

	Gao et al. (2013)<br>https://doi.org/10.1002/bimj.201200034	the median unbiased estimator<br><br>Computed at the point of trial stopping using backward image of the final test statistic (summarised in Gao and Mehta 2013 https://doi.org/10.1002/sim.5847) and inverting test	changes to sample size, timing and frequency of interim analyses, etc)<br><br>Considers non-inferiority hypothesis test followed by a superiority hypothesis test (after rejecting a non-inferiority null hypothesis test)<br><br>Applicable to all types of primary outcomes Symmetric CIs with exact coverage	(assume still available in later versions of EAST)	
**104**	Confidence intervals for confirmatory adaptive two-stage designs with treatment selection<br><br>Bebu et al. (2013)<br>https://doi.org/10.1002/bimj.201200053	Extends the CIs using a conditional likelihood-based approach derived in Bebu et al. (2010, Biom J) to:<br>1) using higher order asymptotics for LRT-based CIs<br>2) more than 2 treatments<br>3) selecting more than 1 experimental treatment at interim (simultaneous confidence region for the means of all selected arms)<br>4) selecting based on both efficacy and safety<br>5) including a control arm<br>6) adjusting for covariates<br>7) binomial endpoints	Two-stage designs with treatment selection<br><br>Conceptually simple and flexible approach, usable e.g. with different selection rules<br><br>Coverage probabilities similar or better than other CI methods	Simulation R code available upon request from corresponding author	Re-analysis of trial data

**105**	Confidence intervals for the selected population in randomized trials that adapt the population enrolled<br><br>Rosenblum (2013)<br>https://doi.org/10.1002/bimj.201200080	Based on computing a minimum factor (c) by which a naïve CI must be expanded asymptotically to have at least desired coverage (e.g., 95%), uniformly over all possible data generating distributions.<br><br>Considered that the ratio of the width of constructed CI relative to the naïve CI to be not more than 1.1 (i.e., inflation of the naïve CI cannot be more than 10%)	Constructing symmetric CIs with a minimum desired width for selected population[s] following a population enrichment design using all data (from selected populations) from both stages for a normally distributed outcome.<br><br>Two-arm two-stage design with 2 disjointed subpopulations.<br><br>They claim CIs are optimal among those that are symmetric.<br><br>May not be feasible to implement for complex decision rules.<br><br>Computationally difficult with increasing number of subpopulations, number of stages and number of treatment arms.<br><br>Considered symmetric CIs<br><br>Doesn't accommodate changes to randomisation ratios and total sample sizes<br><br>Doesn't accommodate designs with flexible rules (e.g., decision rule not prespecified).<br><br>The performance of constructed CIs has not been compared to other possible alternatives.	Provided R code (function) in supplementary material to compute the minimum factor (c) for expanding the naïve CIs (using Equation 5).	No

**106**	Exact inference for adaptive group sequential designs<br><br>Gao and Mehta (2013)<br>https://doi.org/10.1002/sim.5847	Final symmetric stagewise adjusted CIs with exact coverage for one-sided tests around the median unbiased estimator at the point of trial stopping.<br><br>Backward imaging of the observed result is used where the result that was actually observed after additional modifications is transformed into an equivalent result that might have been observed in the original adaptive trial without additional modifications.<br><br>They showed that this backward imaging is unique	Two-arm adaptive group sequential trial. That is, group sequential trials that allow additional adaptations (changes in sample size, number and timing of interim analyses, and decision rules such as error spending functions)<br><br>Method considers early stopping for efficacy. However, they claim that approach can extend to cover both efficacy and non-binding futility stopping.<br><br>One sided superiority hypothesis tests<br><br>Produces symmetric CIs with exact coverage (supported by 3 simulation studies)<br><br>Backward imaging is unique and can handle multiple trial adaptations<br><br>Method developed based on score statistics, so it is applicable to different type of outcomes<br><br>Not applicable to two-sided tests and non-inferiority trials	Implemented in EAST software	Hypothetical example
**107**	Simultaneous confidence intervals that are compatible with closed testing in adaptive designs	Derives confidence regions and lower confidence bounds compatible with decisions of a	Adaptive designs using a two-stage closed testing procedure<br><br>CI compatible with test decision	No	Hypothetical example

	Magirr et al. (2013)<br>https://doi.org/10.1093/biomet/ast035	two-stage closed testing procedure using either a p-value combination test or conditional error approach, by partitioning the parameter space into disjoint regions and applying different tests in each of these.<br><br>Also discusses lower bounds for parameters corresponding to retained hypotheses, lower bounds for the two-stage single-step procedure (Posch et al, 2005, Stat Med), and efficient computation of lower bounds corresponding to rejected null hypotheses.	Not always substantially more informative than test decision		
**108**	Estimation of the relative risk following group sequential procedure based upon the weighted log-rank statistic<br><br>Izmirlian (2014)<br>https://dx.doi.org/10.4310/SII.2014.v7.n1.a4	To construct p-values and a confidence interval which accounts for the sequential design, they use stage-wise ordering and construct an approximate pivotal quantity	Standard GSD to be analysed by weighted log-rank test, with parameter of interest being relative risk.<br><br>Performance of proposed CI not evaluated by simulation	No	No
**109**	A comparison of methods for constructing confidence intervals after phase II/III	Compares different methods for constructing CIs for the	Adaptive seamless phase II/III designs with normal endpoints where the trial always	R, C, and S-Plus code on author's	Hypothetical example

	clinical trials<br><br>Kimani et al. (2014)<br>https://doi.org/10.1002/bimj.201300036	difference between the selected treatment and control, assuming known and common variance across all arms, using simulated data:<br>1) Sampson & Sill (2005, Biom J)<br>2) Stallard & Todd (2005, J Stat Plan Inf)<br>3) Posch et al (2005, Stat Med) which coincides with Magirr et al (2013, Biometrika) for the scenario considered here, and extended to the two-sided case by the authors and use with Sidak and Dunnett corrections, respectively<br>4) Wu et al (2010, Biometrika) which coincides with Neal et al (2011, Stat Med) for the scenario considered here<br>5) naive	continues to the second stage with only the apparently most effective treatment and the control<br><br>All (except naive) CI perform well in most scenarios, with recommendations for which one to choose depending on specific design features and study aims<br><br>They make an interesting suggestion saying that one could also use a hybrid CI consisting of e.g. a Wu et al. or Posch at al. lower bound and a Sampson & Sill upper bound	website but link not working	
**110**	Conditionally unbiased and near unbiased estimation of the selected treatment mean for multistage drop-the-losers trials	Adapts nonparametric bootstrap approach from Pepe et al (2009, Stat Med) to calculate CIs for conditionally unbiased estimators of the selected treatment mean.	Multi-stage drop-the-losers designs with normal endpoint, with focus on 3:2:1 design (3 experimental arms at stage 1, 2 at stage 2, 1 at stage 3)<br>Both approaches provide CIs with nominal coverage in simulations	R code in supplementary material	No

	Bowden and Glimm (2014)<br>https://doi.org/10.1002/bimj.201200245	Uses the profile likelihood approach of Bebu et al (2010, Biom J) to derive CIs for a bias-corrected MLE.	Profile likelihood CI method is much more computationally intensive than bootstrap		
**111**	Testing for efficacy in adaptive clinical trials with enrichment<br><br>Wu et al. (2014)<br>https://doi.org/10.1002/sim.6127	Calculates lower confidence limit for the treatment effect in the selected subpopulation which has coverage probability ≥1-α<br><br>Also provides lower limit for designs with early stopping for superiority or futility	Two-arm enrichment design with sample size reestimation to adaptively select patient subpopulation<br><br>Valid under flexible enrichment rules and sample size reestimation<br><br>Only lower limit available<br><br>Variance assumed to be known (but can be extended to unknown variance case)	No	No
**112**	An evaluation of inferential procedures for adaptive clinical trial designs with pre-specified rules for modifying the sample size<br><br>Levin et al. (2014)<br>https://doi.org/10.1111/biom.12168	Constructs final/exact confidence sets by inverting hypothesis tests using different orderings of the outcome sample space, which are extensions from GSDs to adaptive designs:<br>1) stage-wise (stage of stopping)<br>2) sample mean (MLE)<br>3) signed likelihood ratio statistic<br>4) conditional error, based on Brannath et al. (2009,	Adaptive designs allowing for pre-specified sample size modifications, or stopping for efficacy or futility, based on interim estimates of the treatment effect<br><br>The conditional error-based approach also accommodates unplanned adaptations<br><br>Evaluates CIs in simulations and finds that the LR-based ordering produces CIs with shorter expected length than stage-wise and sample mean ordering.	R code on journal website	No

		Biometrics) and Gao et al. (2013, Stat Med)			
**113**	An Introduction to Practical Sequential Inferences via Single-Arm Binary Response Studies Using the binseqtest R Package<br><br>Kirk and Fay (2014)<br>https://doi.org/10.1080/00031305.2014.951126	Inversion of p-value function based on stagewise ordering.	Single-arm GSD for Bernoulli data.<br><br>Some short notes related to issues around non-binding futility rules or asymmetric stopping rules and what type of CI to present (e.g., if you only have futility stopping do you do a two-sided 95% CI or something else?)	Yes; it is explicitly about an R package.	No.
**114**	Correcting for bias in the selection and validation of informative diagnostic tests<br><br>Robertson et al. (2015)<br>https://doi.org/10.1002/sim.6413	Derives final/exact likelihood-based conditional CIs for a selected classifier's sensitivity (conditional on reaching the validation stage) by adapting the approach of Sill & Sampson (2009, CSDA), and proposes a further tweak to minimise the interval length.<br><br>Also calculates nonparametric bootstrap CIs adapted from Pepe et al. (2009, Stat Med).	Two-stage selection and validation study of binary classifiers for diagnostic tests with arbitrary selection rules and futility thresholds<br><br>Sill-Sampson CIs are conservative<br><br>When tweaked to minimise interval length they become less conservative but computational cost increases substantially	No	Re-analysis of data from screening tool validation study
**115**	Adaptive seamless designs with interim treatment selection: a case study in oncology	Discusses 3 alternative testing procedures and provides simultaneous lower confidence limits for one of them (a	Adaptive seamless phase II/III design<br><br>Properties of CIs not evaluated by simulation	No	No

	Carreras et al. (2015)<br>https://doi.org/10.1002/sim.6407	conservative modification of the Dunnett test) but claims that no informative CIs exist that are consistent with the other 2 procedures when the null hypothesis for the selected treatment is rejected (cf. Strassburger & Bretz, 2008, Stat Med and Guilbaud, 2008, Biom J).			
**116**	Designing issues in confirmatory adaptive population enrichment trials<br><br>Wassmer and Dragalin (2015)<br>https://doi.org/10.1080/10543406.2014.920869	Calculates repeated CIs separately for each subpopulation selected for each stage in enrichment designs using the combination test principle together with a closed testing procedure, using a similar approach as Posch et al (2005, Stat Med).	Adaptive enrichment design with sample size reestimation for different types of endpoints (normal, binary, time-to-event)<br><br>CIs consistent with the test decision if only the subpopulation with the smallest p-value is selected at the first stage<br><br>CIs not generally consistent with the test decision as they do not account for the step-down nature of the closed testing procedure<br><br>CIs may fail to become narrower with increasing sample size for the second and subsequent stages	No	Hypothetical example
**117**	Hypothesis testing for two-stage designs with over or under enrollment<br><br>Zeng et al. (2015)<br>https://doi.org/10.1002/sim.6490	Hypothesis test inversion. Compare to two standard methods of CI calculation for Simon two-stage trials.	Simon two-stage, when there's over and under enrollment.<br><br>Argues hypothesis test inversion approach provides better coverage/width performance.	No.	No.

**118**	Estimation after a group sequential trial<br><br>Milanzi (2015)<br>https://link.springer.com/article/10.1007/s12561-014-9112-6	See Berckmoes (2020); this is basically a precursor to that.		No.	No.
**119**	Sample Size Re-estimation Designs In Confirmatory Clinical Trials—Current State, Statistical Considerations, and Practical Guidance<br><br>https://doi.org/10.1080/19466315.2015.1098564<br><br>Pritchett et al. (2015)	Review article including an overview of CIs and a few recommendations	“The final analysis for a pure bSSR typically requires no adjustment, and therefore the conventional analysis may be performed.”<br><br>“For ubSSR designs, appropriately adjusted confidence intervals should be reported if sample size adaptation occurs. For confidence intervals, the recently developed backward image confidence interval (BWCI) method (Gao, Liu, and Mehta 2013) provides two-sided final/exact confidence intervals for general SSR designs. Gao, Liu, and Mehta (2013) compared this method to repeated confidence interval (RCI) (Mehta et al. 2007) and stage-wise confidence interval (SWCI) (Brannath, Mehta, and Posch 2009). The BWCI has desirable properties, since the RCI method is conservative, and the SWCI method provides only one-sided confidence intervals. Experience with the BWCI method will be needed to assess whether its application to confirmatory clinical trials is accepted by regulators. For confidence	No	No

			intervals, both SWCI and BWCI may be presented as a sensitivity analysis.”		
**120**	Group sequential control of overall toxicity incidents in clinical trials – non-Bayesian and Bayesian approaches<br><br>Yu et al. (2016)<br>https://doi.org/10.1177/0962280212440535	Final/exact single-stage and multi-stage CI based on MLE ordering.	Essentially single-arm Bernoulli, but the focus is on toxicity events<br><br>Overall, both approaches show a higher coverage rate than the confidence level, although the single-stage final/exact confidence interval misses a case. The difference in the average width is minimal, suggesting that the performance of the single-stage final/exact confidence interval is comparable with the multi-stage final/exact confidence interval. Conclusion is that both the single-stage or multi-stage final/exact methods are applicable”	Upon request.	No
**121**	Repeated confidence intervals and prediction intervals using stochastic curtailment under fractional Brownian motion<br><br>Zhang et al. (2016)<br>https://doi.org/10.1080/03610926.2014.919400	Standard RCI methodology; the novelty is in the derivations under fractional Brownian motion.	Stochastically curtailed GSDs<br><br>Proposed RCIs have narrower final confidence intervals and require smaller sample sizes than RCI designs based on classical group sequential designs.	No.	Re-analysis of group sequential trial data
**122**	Efficient confidence limits for adaptive one-arm two-stage clinical trials with binary endpoints	Proposes 3 approaches to construct exact binomial (Clopper-Pearson-type)	Adaptive single-arm two-stage designs with binary endpoints with options to stop for futility or efficacy or to adapt the sample size (Shan et al, 2016, Stat Med)	No	No

	Shan et al. (2017)<br>https://bmcmedresmethodol.biomedcentral.com/articles/10.1186/s12874-017-0297-5	one-sided lower confidence limits for the response rate by ordering the sample space based on:<br>1) p-value<br>2) average response rate at each stage<br>3) asymptotic lower limit<br>And compares them with 3 existing approaches that order the sample space based on:<br>4) average response rate<br>5) likelihood ratio<br>6) score test	Approaches 1 and 3 guarantee at least nominal coverage probability<br><br>Approach 2 can be anticonservative<br><br>Different approaches perform more/less well depending on criterion (simple average length or expected length)		
**123**	Asymmetric inner wedge group sequential tests with applications to verifying whether effective drug concentrations are similar in adults and children<br><br>Hampson et al. (2017)<br>https://doi.org/10.1002/sim.7154	MLE based ordering. Uses a generalisation of a previous paper to define final/exact CI	See title of paper for concise summary of design setting (note this means it's an equivalence test).<br><br>CIs are compatible with test decision in theory, but some deviation (~1%) seen in simulation study<br><br>The coverage of 90% CIs remains close to the nominal level in all scenarios, with deviations within ± 5%.	No.	No
**124**	Comparison of conditional bias-adjusted estimators for interim analysis in clinical	CMAE, CUMVUE, CMUE, and weighted estimator compared	Standard GSD with survival (Weibull) data.	No.	Re-analysis of group sequential

	trials with survival data<br><br>Shimura et al. (2017)<br>https://doi.org/10.1002/sim.7258	in terms of conditional/unconditional bias/RMSE.<br>To get associated CIs, use non-parametric bootstrapping.	Recommended CUMVUE for trials that don't terminate early and CMAE for those that do.		trial data
**125**	Estimation in multi-arm two-stage trials with treatment selection and time-to-event endpoint<br><br>Brückner et al. (2017)<br>https://doi.org/10.1002/sim.7367	Uses a bootstrap resampling approach similar to Bowden & Glimm (2014, Biom J), conditional on the selection in the original dataset, to obtain CIs for shrinkage and Stallard-Todd estimators.	Two-stage multi-arm design with a common control and time-to-event endpoint where the best treatment is selected at interim<br><br>Bootstrap cannot properly account for the variability introduced by the selection process as the selection in a resampled dataset may be different from the original dataset	No	Re-analysis of multi-arm trial data
**126**	Test-compatible confidence intervals for adaptive two-stage single-arm designs with binary endpoint<br><br>Kunzmann and Kieser (2018)<br>https://doi.org/10.1002/bimj.201700018	Constructs a Clopper-Pearson-type CI for the response rate using an ordering of the sample space derived from a test-compatible point estimator (Kunzmann & Kieser, 2018, Stat Med).<br><br>A direct optimisation approach minimising the mean width only gives marginally tighter CIs but is computationally intensive and does not guarantee control of the coverage probability.	Adaptive single-arm two-stage designs with binary endpoints with options to stop for futility or efficacy or to adapt the sample size<br><br>CI always compatible with test decision<br><br>Assures nominal coverage probability<br><br>May be unnecessarily wide	Julia package 'BinaryTwoStageDesigns' and supplementary material	No
**127**	On the sample mean after a group	This is a precursor to		No	No

	sequential trial<br><br>Berckmoes et al. (2018)<br>https://doi.org/10.1016/j.csda.2018.03.016	Berckmoes (2020), with less general results.			
**128**	Exact Inference for Adaptive Group Sequential Designs<br><br>Mehta et al. (2019)<br>https://link.springer.com/chapter/10.1007/978-3-319-67386-8_10	Presents highlights from Gao and Mehta (2013), who proposed the backward image confidence interval (BWCI)	Simulation results demonstrate correct coverage<br><br>RCI is increasingly conservative (as true treatment effect increases) in comparison, and is extremely asymmetric. In contrast, The BWCI interval excludes the true value for θ with 0.025 probability symmetrically from below and above<br><br>Theory is expressed in terms of score statistics and so is applicable to all types of efficacy endpoints including normal, binomial and survival endpoints and model-based endpoints derived from contrasts of regression parameters and estimated by maximum likelihood methods.	No	No
**129**	On asymptotic normality in estimation after a group sequential trial<br><br>Berckmoes (2020)<br>https://doi.org/10.1080/07474946.2020.1826784	Demonstrate that in many settings ordinary sample mean is asymptotically normal after a standard GSD. Use this to suggest naive CIs are often fine.	Standard two-arm GSD with normal data.<br><br>There are conditions on the result in relation to the stopping rules and the value of the outcome mean.	No	No

**130**	Estimation of treatment effects following a sequential trial of multiple treatments<br><br>Whitehead et al. (2020)<br>https://doi.org/10.1002/sim.8497	Provides approximate CIs via a Rao-Blackwellisation approach based on expected value of an unbiased estimate computed at the first interim analysis (thus unaffected by adaptation rules), conditional on sufficient statistics computed at the end.<br><br>Determines test statistic and its SE by 'reverse simulation': based on the final data, replicate realisations of samples at earlier interim analyses are generated, only keeping those consistent with continuing to the observed end of the trial	Complex sequential or adaptive designs involving multiple arms and a binary endpoint<br><br>Applicable to ADs with complicated designs such as multiple treatments and flexible adaptive features e.g. multi-arm design without a designated control where all pairwise comparisons are of interest<br><br>For simpler (e.g. two treatment) designs, methods based on orderings of the final sample space provide more accurate CIs and are less computationally intensive<br><br>CIs tend to be conservative	SAS code as supplementary material	Hypothetical example
**131**	Pharmacokinetics and Pharmacodynamics of a Proposed Pegfilgrastim Biosimilar MSB11455 Versus the Reference Pegfilgrastim Neulasta in Healthy Subjects: A Randomized, Double-blind Trial<br><br>Lickliter (2020)<br>https://doi.org/10.1016/j.clinthera.2020.05.020	"Adjusted and repeated" CIs	Group-sequential AB/BA crossover trial for equivalence.	No.	This is an example of a trial that computed adjusted CIs, but there is very little detail given.

**132**	Exact confidence limits after a group sequential single arm binary trial<br><br>Lloyd (2021)<br>https://doi.org/10.1002/sim.8909	Generalises exact method of Jennison and Turnbull (1983) by placing their method within the theory of exact confidence limits ('Buehler limits') which gives an optimal (smallest possible paper limit) once a ranking function has been chosen<br><br>Proposes different ranking functions (i.e. ordering of the sample space), the first based on the upper limit of the Clopper and Pearson (CP) confidence limit and the second based on the approximate likelihood ratio (LR) upper limit<br><br>Also explores other ranking functions that are not based solely on the minimal sufficient statistic (i.e. violating the sufficiency principle), such as one based on inverting the Fisher's combination test (FI)	Single-arm group sequential trial with binary outcomes<br><br>Gives simple condition to assess whether a confidence limit is compatible with the test<br><br>In terms of size of the exact upper/lower limits (smaller/larger being better), the CP and LR are better, although the differences are often quite modest<br><br>FI based methods have a uniform improvement but the advantage is practically very small, and methods are computationally difficult<br><br>If limits and test results conflict, can modify the limits to make them compatible with the test or conclude that the test itself needs redefining.	Yes, there is an R package (CLAST)	No

**133**	Group sequential methods for the Mann-Whitney parameter<br><br>Nowak et al. (2022)<br>https://doi.org/10.1177/09622802221107103	Show test statistics follow canonical joint distribution, allowing application of standard repeated confidence interval methodology.<br>Test inversion used for Brunner-Munzel.<br><br>Exploits asymptotic canonical joint distribution to then use methodology for repeated confidence intervals	Standard two-arm group sequential setting, but focused on Wilcoxon-Mann-Whitney and Brunner-Munzel tests.<br><br>Group sequential trials with with nonparametric endpoint (relative effect/Mann-Whitney parameter)<br><br>No simulation study of coverage	R code available	Re-analysis of group sequential trial data
**134**	Conditional information and inference in response-adaptive allocation designs<br><br>Lane (2022)<br>https://doi.org/10.1002/sim.9243	Proposes a conditional bootstrap CI procedure consistent with the conditional MLE (conditional on the observed treatment sample sizes).	Response-adaptive design with binary endpoints<br><br>In many cases the conditional bootstrap CIs are narrower than relevant unconditional CIs (because conditional can be greater than unconditional information)	No	Re-analysis of RAR trial data
**135**	A simple solution to the inadequacy of asymptotic likelihood-based inference for response-adaptive clinical trials<br><br>Baldi Antognini (2022)<br>https://link.springer.com/article/10.1007/s	Proposes a re-scaling of the target (optimal allocation) to overcome inadequacies of likelihood-based inference and asymptotic CIs.	Response adaptive randomisation designs with different types of endpoints<br><br>Properties of CIs not evaluated by simulaiton	No	No

	00362-021-01234-3				
**136**	A new inferential approach for response-adaptive clinical trials: the variance-stabilized bootstrap<br><br>Baldi Antognini (2022)<br>https://link.springer.com/article/10.1007/s11749-021-00777-9	Proposes a new inferential strategy for response adaptive designs which combines a variance stabilising transformation with a parametric bootstrap method and allows for computation of bootstrap CIs.	Response adaptive randomisation designs with different types of endpoints<br><br>Good empirical coverage of proposed CIs demonstrated by simulation (although sometimes conservative)<br><br>“The suggested bootstrap approach turns out to provide reliable confidence intervals in terms of both empirical coverage and interval width”	No	No
**137**	A simulation-based comparison of estimation methods for adaptive and classical group sequential clinical trials<br><br>Nelson et al. (2022)<br>https://doi.org/10.1002/pst.2188	Repeated CI, Backward Image CI (for adaptive GSD case), Stagewise Adjusted CI (for standard GSD case), MUE.<br><br>The methodology is not novel here; the contribution is a comparison of the performance of several CIs (in terms of width, coverage, symmetry).	Adaptive and standard GSDs<br><br>“When possible, one should prefer the BWCI for estimation following an adaptive group sequential trial. In addition to providing exact and symmetrical coverage … it has the desirable property of being consistent with the hypothesis test… However, if the BWCI cannot be computed (for example due to overruns, not following efficacy boundary rules, or during an interim look), the RCI should be preferred to the naïve CI because the naïve CI is somewhat unpredictable - one cannot be sure that the naïve CI is providing appropriate coverage and may not remain consistent with the	Yes; East and R code available in supplementary materials.	No

			corresponding hypothesis test.“		
**138**	Exact confidence limits compatible with the results of a sequential trial<br><br>Lloyd (2022)<br>https://doi.org/10.1016/j.jspi.2021.07.014	Similar paper to Lloyd (2021), covers the same theoretical considerations with a focus on single-arm trials with option for early stopping for efficacy or futility (e.g., Simon’s two stage design)<br><br>Final/exact one-sided CI constructed (at the end of the trial) using a ranking function to rank all elements of the sample space. Can be constructed without relying on a p-value or test statistic.	Gives a formal definition of what it means for final/exact confidence limits to be compatible with a (sequential) test and provided a simple and intuitive condition for compatibility<br><br>Shows that the framework of Liu and Anderson (2008) gives CIs that are conservative by construction (since is not a Buehler limit), does not allow for stopping for futility and are potentially computationally difficult<br><br>“When limits and test results conflict, we have two choices. We have explained how to modify the limits to make them compatible with the test. Alternatively, we might conclude that the test itself needs redefining.”<br><br>No guarantee that the CI will agree with the test decision (proposed a simple sufficient condition on the ranking function or modification of the sample space ordering to ensure compatibility of the CI and test	No	No

			result)<br>There are many possible ranking functions (and these may have different properties or leads to different results)		
**139**	Evaluating group-sequential non-inferiority clinical trials following interim stopping – the HIV Prevention Trials Network 083 Trial<br><br>Hanscom et al. (2022)<br>https://doi.org/10.1177/17407745221118371	Example of applying final/exact confidence interval (based on MLE ordering) in practice	Group-sequential non-inferiority trial with 4 interim analyses and O'Brien Fleming boundaries	No, but mentions the RCTDesign R package and SAS procedures (PROC SEQDESIGN and PROC SEQTEST)	Methods applied in practice to this trial
**140**	Properties of a Confirmatory Two-Stage Adaptive Procedure for Assessing Average Bioequivalence<br><br>Osterdal et al. (2023)<br>https://doi.org/10.1080/19466315.2023.2223561	Inverts overall p-values obtained using combination test (Brannath et al. 2002)	By construction, the p-value based confidence interval is aligned to the decision regarding the two one-sided hypotheses<br><br>Properties of coverage of CIs is only asymptotic<br><br>Empirically, coverage probabilities can be very conservative (well above the nominal)<br><br>In some settings there can be instances when the lower bound is above the upper		

			bound		
**141**	A seasonality-adjusted sequential test for vaccine safety surveillance<br><br>Shen et al. (2023)<br>https://doi.org/10.1111/biom.13829	Final/exact CI by inverting hypothesis test, but p-value function calculated by bootstrap/resampling<br><br>Constructs one-sided CIs	Survival data assuming Poisson distribution, parameter of interest is the relative risk<br><br>Simulation results show that the empirical coverage is close to the nominal<br><br>Empirical average of upper ends of the 95% CI was "fairly close" to zero when the true parameter was equal to zero, suggesting that the resulting confidence interval was an informative decision	States R code is available but could not find the code online	No
**142**	The population-wise error rate for clinical trials with overlapping populations<br><br>Brannath et al. (2023)<br><br>https://doi.org/10.1177/09622802221135249	Proposes simultaneous confidence intervals compatible with a multiple test procedure that controls the population-wise error rate. These CIs are formed by inverting the (asymptotic) pivotal test statistics, taking into account the (asymptotic) joint distribution.	Framework applicable for clinical trials with multiple, overlapping populations e.g. basket or umbrella trials<br><br>Cis control the average simultaneous coverage probability (i.e. average the strata-wise coverage probability over the entire population).	No	No
**143**	Adaptive Multiple Comparison Sequential	Follows same method as in	Essentially a MAMS design: group	DACT software	No

	Design (AMCSD) for clinical trials<br><br>Gao and Li (2024)<br>https://doi.org/10.1080/10543406.2023.2233590	Gao et al. (2013) for adaptive group sequential trials	sequential stopping boundaries to test efficacy of multiple treatments with sample size re-estimation<br><br>Proposed confidence interval is wider than the final/exact interval (since the latter requires knowledge of the true parameters)<br><br>Upper and lower confidence limits are conservative in terms of coverage		
**144**	Confidence intervals for odds ratio from multistage randomized phase II trials<br><br>Cao and Jung (2024)<br>https://doi.org/10.1002/sim.10073	Final/exact group sequential CIs based on stage-wise ordering	Group sequential design based on Fisher's exact test.<br><br>Conditional coverage probabilities of exact test maintains the nominal level	No	Re-analysis of group sequential trial data
**145**	Point estimation, confidence intervals, and P-values for optimal adaptive two-stage designs with normal endpoints<br><br>Meis et al. (2024)<br>https://doi.org/10.1002/sim.10020	Final/exact CIs based on different families of orderings of the sample space (MLE, likelihood ratio test, score test, Neyman-Pearson test, stage-wise combination function)<br><br>Repeated confidence intervals	Two stage optimal single-arm trials with normally-distributed endpoints<br><br>Naive construction can lead to CIs that are not single intervals<br><br>The associated testing procedure of the confidence interval may not necessarily agree with the primary testing procedure of	Yes<br>See https://github.com/jan-imbi/adestr and the R package *adestr*	No

		also derived	the trial. This is only guaranteed for the stage-wise combination function ordering. The confidence interval based on the score test ordering has particularily bad agreement with the test decision<br><br>All of the confidence intervals induced by some sample space ordering have a coverage that is indistinguishable from the nominal. For the naive confidence interval, this is not guaranteed. However, the naive confidence interval has the lowest average width of all of the compared estimators.<br><br>The repeated confidence interval is generally wider than the other confidence intervals.<br><br>Neyman-Pearson test ordering performs very badly		
**146**	Conditional bias adjusted estimator of treatment effect in 2-in-1 adaptive design<br><br>Bai et al. (2024)<br>https://doi.org/10.1080/10543406.2024.2359147	CI constructed around conditional bias adjusted estimator using normal approximation (with conditional variances calculated using the multivariate delta method)	Trial setting is ‘2-in-1’ adaptive design, which allows switching from a Phase II trial into a Phase III trial<br><br>For a reasonable range of cutoff values, the conditional coverage rates are close to the nominal level. However, both under- and over- coverage can occur, with undercoverage if the cutoff value is large or	No	No

			when the true treatment effect is small		
**147**	Statistical Inference for a Two-Stage Adaptive Seamless Design Using Different Binary Endpoints<br><br>Ishii et al. (2025)<br>https://doi.org/10.1002/sim.70003	Final CIs are constructed by inverting p-value functions based on different (adjusted) estimators	Two-stage adaptive seamless designs with selection at interim based on short-term binary endpoint and final analysis comparing long-term binary endpoints<br><br>Coverage probability general close to the nominal 95% when using the 'mid' p-value function, whereas noticeably conservative coverage is seen when using the 'exact' p-value function	Yes, R code available at https://github.com/rtishii	Hypothetical examplel

Additional relevant papers cited by papers in the above table

**Paper title & authors**	**Brief description of the methodology**	**Trial context and Advantages / limitations**	**Code/software available?**	**Case studies?**
On the estimation of the binomial probability in multistage clinical trials<br><br>Jung and Kim (2004)<br>https://doi.org/10.1002/sim.1653	Uses same methodology as Jennison and Turnbull (1983) to produce an final/exact CI based on stochastic ordering by the magnitude of the UMVUE	Group sequential trial designs with binary responses<br><br>Final/exact CI is wider than the standard Clopper-Pearson CI, as it takes into account the GS design used<br><br>No simulation study looking at coverage	No	No
Interim analysis: the repeated confidence interval approach<br><br>Jennison and Turnbull (1989)<br>https://www.jstor.org/stable/2345448	Presents the general principles underlying the construction of RCIs and describes how they can be used in reaching a decision to terminate a study early<br><br>RCIs are constructed by inverting the standardised test statistic (appropriate for the type of response data being monitored)	Group sequential trials with a variety of types of response data (including survival data, categorical data, normal responses with unknown variance and multivariate normal observations), and with unequal increments in information between analyses<br><br>Properties of RCIs strongly depend on which group sequential test is used<br><br>RCIs retain their overall confidence level whether or not a stopping rule is used. They may be calculated as and when the	No	No

		investigator pleases, at any stage of the study.<br><br>Question (from Peter Armitage): “Is it necessary to have a probabilistic guarantee that *all* the confidence statements are correct? Should we not be content if *most* were correct? Even though we might wish to make provisional statements during the course of a study, more importance will usually attach to the terminal statement, and I am doubtful about the wisdom of introducing conservatism here merely to ensure a guarantee over the whole set of intervals.”<br><br>Parmar et al. reply: “The type of RCI to be used needs to be specified during the design of the trial. The authors suggest that this prespecified RCI can be used whatever the stopping rule employed. This could clearly lead to different conclusions from the stopping rule and the RCI methods. Thus, in practice, we question whether the RCIs can be divorced from the actual stopping rule employed. Since each RCI has a naturally related stopping rule it would seem appropriate to employ this one.”<br><br>Casella reply: “One of the major strengths of		

		the authors' approach is that their interim analyses produce acceptable interim inferences, i.e. acceptable inferences conditional on stopping."<br><br>Some replies express concern that RCIs (for normal response with known variance) are symmetric around the sample mean, which is known to be biased away from the null		
On the determination of one-sided confidence limits in adaptive interim analysis<br><br>Wassmer and Lehmacher (1997)<br><br>Proceedings der 42. Jahrestagung der GMDS, Miinchen MMV Medizin. 1997;340-344.	RCIs for adaptive GSD designs, extension of the methodology of Jennison and Turnbull (1989)	Two-stage adaptive GSD using combination test and conditional power approach, for normally distributed variables<br><br>RCIs wider than standard, differences are smallest for moderate effect sizes<br><br>RCIs based on conditional power approach only asymptotically valid (but only slightly anti conservative in terms of coverage), whereas RCIs based on combination test are exact	No	No
Confidence intervals for a binomial parameter following a multistage test with application to MIL-STD 105D and medical trials.	Proposes CIs upon termination of group sequential trial, based on inverting test statistic with stagewise ordering	Group sequential trials with binary responses<br><br>"The sequential confidence intervals calculated are often close to the fixed	No (computer program available upon request…)	No

Jennison and Turnbull (1983)<br>https://www.jstor.org/stable/1267726		sample size interval based on the same number of observations and successes; in some cases there are large enough differences to make it worthwhile to compute the proper sequential confidence interval. When choosing a sequential sampling plan it is useful to consider what a resulting confidence interval might be; a good plan should always produce an interval that is precise enough to allow a decision to be reached”		
Confidence Intervals Following Group Sequential Testing: Conditioning on Stopping Time, Masters Thesis, Cornell University, Ithaca, NY.<br><br>Ohman (1996)<br>https://ecommons.cornell.edu/bitstream/handle/1813/31927/BU-1331-M.pdf;jsessionid=BB1AEEE45BD4C2C96510DA1F285BAB59?sequence=1	Presents confidence intervals which are correct when conditioning on the subset of data such that a trial stopped at a particular analysis.<br><br>Inverts test statistic, using stochastic ordering conditional on the stopping time and the direction of the observation away from the null	Group sequential designs with normally distributed outcomes (and known variance)<br>“Although these intervals may not be practical, they do have very desirable properties for observations which are highly unusual (given any value of the mean).”<br>The conditional intervals behave well when an improbable result is observed. However, they become very wide (tending to infinity) when close to the boundary.<br>When the null hypothesis that the mean is equal zero has been rejected and the value is close to the boundary, the null value will be included in the conditional interval<br><br>Conditional coverage probabilities are used as a way to compare the sample mean, stagewise, and repeated confidence intervals. “However, none of these intervals	No	No

		outperforms the others when conditioning on stopping time."<br>All previously proposed unconditional orderings reduce to this ordering after conditioning on the stopping time.<br>Unconditional CIs can have highly variable conditional coverage, ranging from 0 to 1		
Estimation following sequential tests.<br><br>Siegmund (1978)<br>https://doi.org/10.2307/2335213	CIs formed by inverting test statistic, using ordering of sample space	Sequential trials with normally distributed outcomes<br>Based on asymptotic distribution<br>Properties of CIs not evaluated by simulation	No	No
Unbiased Estimation of Selected Treatment Means In Two-Stage Trials<br><br>Bowden and Glimm (2008)<br>https://doi.org/10.1002/bimj.200810442	Constructs CI by assuming approximate normality of UMVCUE and that the variance is maximised when all treatment means are the same	Two-stage drop-the-loser design with normally distributed outcomes<br><br>When the true difference between each treatment is 0 coverages are slightly above or below the nominal level<br><br>When there is a small difference between the true treatment means, all of the estimate's coverage probabilities appear at, or above, their nominal 95% level.<br><br>Confidence intervals based on the MLE are narrower than those based on the UMVCUE,	No	No

		but their alpha-level control is clearly suspect.		
Estimation after sequential testing: A simple approach for a truncated sequential probability ratio test<br><br>Woodroofe (1992)<br>https://doi.org/10.1093/biomet/79.2.347	Uses asymptotic results to construct approximate standard normal pivot which can then be used to form CI	Sequential trial with normally distributed outcomes<br><br>The simulations suggest that the approximation is quite accurate, but can fail in certain cases	No	No
Corrected confidence intervals after sequential testing with applications to survival analysis<br><br>Coad and Woodroofe (1996)<br><br>https://doi.org/10.1093/biomet/83.4.763	Uses asymptotic results to construct approximate standard normal pivot which can then be used to form CI	Sequential trials with observations from a one-parameter exponential family<br><br>The simulations suggest that the approximation is quite accurate, but can fail in certain cases		
Adaptive Sample Size Calculations in Group Sequential Trials<br><br>Lehmacher and Wassmer (1999)<br>https://doi.org/10.1111/j.0006-341X.1999.01286.x	Extends RCI methodology of Jennison and Turnbull (1989)<br><br>Based on inverse-normal combination method	Adaptive group sequential trials, which allow data-driven sample size reassessments. Normally distributed observations with known variance<br><br>"RCIs are valid whatever stopping criterion is employed and hence are different from	No	Hypothetical example

		confidence intervals following a group sequential test."<br><br>Performance of RCIs not evaluated by simulation		
Confidence intervals for secondary parameters following a sequential test<br><br>Whitehead et al. (2000)<br>https://doi.org/10.1111/1467-9868.00260	Woodroofe's pivotal method is used to obtain confidence intervals in a generic setting concerning location parameters of bivariate normal responses with known covariance structure. One is treated as the primary parameter of interest for which a sequential test is conducted; the other is secondary, being estimated only when the sequential trial stops. Using approximations to normality, the results can be applied asymptotically in a variety of settings	Sequential tests with variety of outcome types, including binary and normal<br><br>Simulation results show CIs have coverage close to nominal<br><br>A crucial assumption which is needed for this methodology to work is that information accumulates in proportional fashion about both the primary and the secondary parameters.Many situations involving survival data contradict this requirement	No	No
Statistical inference for extended or shortened phase II studies based on Simon's two-stage designs<br><br>Zhao et al. (2015)<br>https://doi.org/10.1186/s12874-015-0039-5	It constructs final/exact confidence intervals for Simon's two-stage designs when there are study deviations from the planned sample size. It uses likelihood-based ordering of the sample space to derive exact analytical confidence intervals. Simulations are performed to assess the validity of analytical derivations and to compare the results with alternative methods.	It focuses on early phase trials and very narrow scenarios of unplanned changes to sample size in Simon's two-stage design.<br><br>It provides shorter coverage and an unbiased CIs in most scenarios in the simulation studies presented.	No	Re-analysis of Simon's two-stage trial data

An exact method for analysis following a two-stage phase II cancer clinical trial<br><br>Jovic and Whitehead (2013)<br>https://doi.org/10.1002/sim.3837	This article proposes a final/exact CI which can be applied for a single-arm phase II trial with a planned interim analysis. The approach is presented using trials with binary outcomes. It compares the coverage of the final/exact method against the approximate or asymptotic approach which based on the score (and Z-test) statistic.	Single arm two stage phase II trial with binary outcomes.<br><br>Provides a better coverage than the asymptotic approach in some scenarios.<br><br>Can not be easily applied to two-arm comparative designs, and calculations for more than two stages can be complex.	No	No
Interval estimation of binomial proportion in clinical trials with a two-stage design<br><br>Tsai et al. (2008)<br>https://doi.org/10.1002/sim.2930	It proposes interval estimation for a two-stage phase II trial with a binary outcome. It compares the final/ exact confidence intervals against two asymptotic interval estimators, Wald and score, in the context of Simon's two-stage design. The article explores several other approaches as well, including those of Clopper–Pearson and Sterne as well as approaches with continuity correction.	It is mainly focused on Simon's two-stage design. According to the mean coverage probability and expected interval width, that with continuity correction performs better than that without continuity correction in terms of coverage probabilities, especially when stage 1 sample size is considerably larger than stage 2 for Simon's minimax designs.<br>Analytically, it is quite complex - particularly for multi-stage designs. The gain in terms of efficiency seems to be moderate, compared with other less complicated asymptotic approaches.	No	Re-analysis of (what was then) an ongoing trial
Sequential estimation for covariate-adjusted response-adaptive designs	This article proposes a sequential procedure for estimating treatment effect under a general	The procedure can be applied to other generalised linear models - although the article explores the issues in the context of a	No	No

Chang and Park (2013)<br>https://doi.org/10.1016/j.jkss.2012.06.001	covariate-adjusted response-adaptive designs.<br><br>It derives asymptotic confidence sets for the multi-arm response-adaptive trial designs. It also studies the coverage probability both analytically and using simulations.	binary outcome.<br>The required sample size can become too large if the number of covariates are large. There are also technical/statistical issues when covariates are correlated. Finally, the procedure would require unblinding of the trial results, which can invoke selection bias.		
Confidence intervals for a binomial parameter based on multistage tests<br><br>Duffy and Santner (1987)<br>https://doi.org/10.2307/2531951	Use a fine grid of p_i to find the minimal set A(p_i) whose joint mass function of stopping stage and number of successes for outcomes consistent with stopping has nominal level.<br><br>Uses ordering based on total number of responses	Trial context is for binary outcomes and a (pre-planned) sequential test<br><br>CIs have uniformly shorter total length, nearly uniformly shorter expected length and closer to nominal probability of coverage<br>than Clopper-Pearson based intervals	No	No
Confidence limits for probability of response in multistage phase II clinical trials<br><br>Atkinson and Brown (1985)<br>https://doi.org/10.2307/2531294	For binary outcomes.<br><br>Uses Clopper and Pearson to construct confidence intervals based on a recursive formula by Schultz 1973 for the number of responses at a given stage.<br><br>Uses ordering based on total number of responses	Trial context is for binary outcomes and a (pre-planned) sequential test<br><br>CIs give nominal coverage.	Fortran code from the authors	No
Exact confidence limits for the probability of response in two-stage designs	Two separate approaches both based on Clopper-Pearson lower bound	Trial context is for binary outcomes, pre-planned 2-stage trial with futility stopping only	No	No

Shan (2018a)<br>https://doi.org/10.1080/02331888.2018.1469023	One based on the ordering of the total number of responses (unclear how different from Atkinson and Brown above) and one on ordering of p-value.	Both CI approaches give nominal coverage. Lower limits are always lower or equal to limits based on inverting (which do not always control coverage)		
Exact confidence limits for the response rate in two-stage designs with over- or under-enrollment in the second stage<br><br>Shan (2018b)<br>https://doi.org/10.1177/0962280216650918	Lower CI limits based on Clopper-Pearson lower bound using p-value ordering and also total number of responses.<br><br>Unclear what is different to paper above in terms of methodology	Trial context is for binary outcomes, pre-planned 2-stage trial with futility stopping only<br><br>Both CI approaches give nominal coverage	No but promises an R package	No
Interval and point estimation in adaptive phase II trials with binary endpoint<br><br>Nhacolo and Brannath (2019)<br>https://doi.org/10.1177/0962280218781411	CIs constructed at trial end. A modified stagewise ordering of the sample space that takes into account the conditional error function and adaptation rule is used. A CI is then obtained by inverting hypothesis tests.	Single arm two stage GSD with binary outcome, pre-specified adaptation rules where the sample size for stage 2 is a function of the number of responses from stage 1 (i.e., via a conditional error function)<br><br>Methods can be extended to flexible designs (i.e., doesn't require prespecification)<br><br>Modified stagewise ordering to address inconsistency with decision rule in some cases when stagewise ordering is used. This is achieved by taking into account the conditional error function and adaptation rule in the stagewise ordering in the scenario when the trial continues to stage 2.	No<br><br>But stated that the R code is available from the authors upon request	No

		Different functions to modify stagewise ordering are proposed.<br><br>Based on extensive simulations, they concluded that one-sided CIs from proposed methods have good coverage.		
On construction of smallest one-sided confidence intervals for the response rate in adaptive two- or multi-stage designs<br><br>Wang et al. (2020)<br>https://doi.org/10.1177/0962280219873359	Final/exact lower one-sided CI for response rate at the end of the trial with a guaranteed confidence level.<br><br>Derived using a rank function that is based on the Clopper-Pearson lower confidence limit.<br><br>Proposed two methods with or without sufficiency principle: one based on reduced sample space and another on the original sample space.	Single arm multi-stage design with a binary outcome. Goal is to construct a lower one-sided CI for the response rate.<br><br>Early stopping option for efficacy or futility. Started with a 2 stage design but the concept extended to multi-stage. Proposed final/exact CIs seem to perform quite well<br><br>May make an incorrect inference of response rate being 100% in certain sample points (meaningless conclusions) but suggesting fixing this by adding an imaginary point to the sample space.	No	Re-analysis of data from a Simon's optimal two stage design
What inference for two-stage phase II trials?<br><br>Porcher and Desseaux (2012)<br>https://doi.org/10.1186/1471-2288-12-117	Proposed 2 methods for constructing two-sided CI at the end of the trial:<br>1) mid-p approach – an extension of stagewise ordering CI	Simon's two stage design<br><br>Reviewed and compared the properties of different methods for constructing two-sided CIs (including naïve exact CI).<br>● Final/exact clopper-pearson CIs using stagewise ordering	No	No

	2) Conditional mid-p (mid-p CI using conditional distribution)<br><br>Compared these with other methods proposed in the literature	• Mid-p CIs – an extension of stagewise CIs<br>• Conditional mid-p – modification of mid p-CI but using the conditional distribution of outcomes at stage 2<br>• Conditional final/exact<br>• Conditional score<br><br>They claimed that mid-p CIs performed better than final/exact CIs for both conditional or unconditional inference in most settings<br><br>For conditional inference, they recommended the conditional mid-p or conditional score CIs		
Underestimation of treatment effects in sequentially monitored clinical trials that did not stop early for benefit<br><br>Marschner and Schou (2019)<br><br>https://doi.org/10.1177/0962280218795320	Replace the MLE with the conditional MLE and determine the width of the CI using an estimate of the standard error of the conditional MLE	Group sequential design, results all conditional on trials not stopping early<br><br>The conditional confidence interval has very good coverage probability across a range of scenarios, including those where the unconditional confidence interval has very poor performance.<br><br>Empirical coverage of the value 0 by the confidence interval agreed with rejection or non-rejection of the null hypothesis using the sequential test<br><br>Restricted conditional confidence interval of	Yes (R code provided in supplementary materials)	Re-analysis of group sequential trial data

		Fan and DeMets (2006) is narrower than the other confidence intervals, “but can have a tendency to be highly asymmetric around the conditional MLE for studies that did not stop early. Such behaviour is difficult to interpret and may suggest that this method is more applicable for studies that did stop early, where the information loss due to conditioning tends to be greater.”		
Estimation of the treatment effect following a clinical trial that stopped early for benefit<br><br>Marschner et al. (2022)<br>https://doi.org/10.1177/09622802221122445	Replace the MLE with a penalised MLE (pMLE), which is a compromise between the MLE and conditional MLE.<br><br>The CI is calculated using a (conditional) bias-corrected bootstrap procedure on the pMLE values.	Group sequential design, results all conditional on trials stopping early.<br><br>Coverage of pMLE CIs were between 92-94%, higher than for conditional MLE CI<br><br>pMLE avoids the undesirable property of long-left hand tail of conditional MLE CI (that often also covers zero)<br><br>The coverage probability of pMLE confidence interval will be zero under the null, because it has been deliberately constructed to always exclude the null	Yes (R code provided in supplementary materials)	Re-analysis of group sequential trial data
Estimation and confidence intervals after adjusting the maximum information	CI is derived by inverting the adaptive test statistic	Two-arm trial setting with single interim where investigator can adjust the maximum	No	No

Lawrence and Hung (2003)<br>https://doi.org/10.1002/bimj.200390001		information level<br><br>CI is compatible/consistent with test decision<br><br>The coverage probability of the adaptive CI is very close to 0.95 in each of the scenarios studied.		
Point and interval estimation in two-stage adaptive designs with time to event data and biomarker-driven subpopulation selection<br><br>Kimani et al. (2020)<br>https://doi.org/10.1002/sim.8557	Follows methodology of Magirr et al. (2013) to derive simultaneous CIs that are compatible with the testing decision	Two-stage adaptive clinical trials with subpopulation selection<br><br>CIs can be uninformative<br><br>“In all scenarios, as desired, the coverage probabilities for the confidence regions are at least 95%. The confidence regions are not symmetric but the probabilities that at least one upper bound is less than the true value are below the desired 2.5%. However, these probabilities tend to be very small compared to the target 2.5%. This is partly due to the non-informative upper bounds.”	Yes (R code provided in supplementary materials)	Re-analysis of adaptive enrichment trial data
Group sequential trials revisited: Simple implementation using SAS<br><br>Whitehead (2010)	Final/exact CIs based on stagewise ordering	No simulation results presented	Yes (SAS code provided in article)	No

https://doi.org/10.1177/0962280210379036				
A unifying family of group sequential test designs<br><br>Kittelson and Emerson (1999)<br>https://doi.org/10.1111/j.0006-341x.1999.00874.x	No specific CIs proposed but provides a unifying framework for group sequential designs	A family of group sequential designs that unifies previous approaches and allows continuous movement among the previous categories. The approach facilitates the process of tailoring the design to address important clinical issues. The unified family of designs is constructed from a generalization of a four-boundary group sequential design in which the shape and location of each boundary can be independently specified. Methods for implementing the design using errorspending functions are described.	The design family is implemented in the RCTDesign R package and SAS procedures<br>(PROC SEQDESIGN and PROC SEQTEST)	Hypothetical example
Flexible implementations of group sequential stopping rules using constrained boundaries<br><br>Burington and Emerson (2003)<br>https://doi.org/10.1111/j.0006-341X.2003.00090.x	No specific CIs proposed but provides a general framework for group sequential designs where the number and timing of interim analyses are unknown	Group sequential designs where the number and timing of interim analyses are not precisely known at the time of trial design, and thus the implementation of a particular stopping rule must allow for flexible determination of the schedule of interim analyses.<br><br>Considers the use of constrained stopping boundaries in the implementation of stopping rules.	The design family is implemented in the RCTDesign R package and SAS procedures<br>(PROC SEQDESIGN and PROC SEQTEST)	Hypothetical example

		This approach is evaluated on various scales for the test statistic. When implemented on the scale of boundary crossing probabilities, this approach is identical to the error spending function approach of Lan and DeMets (1983).		
